# Design of five two-dimensional Co-metal-organic frameworks for oxygen evolution reaction and dye degradation properties

**DOI:** 10.3389/fchem.2022.1044313

**Published:** 2022-11-10

**Authors:** Chuanbin Fan, Xiaoyin Zhang, Feng Guo, Zhiyong Xing, Junli Wang, Wanying Lin, Jie Tan, Guimei Huang, Ziao Zong

**Affiliations:** ^1^ School of Laboratory Medicine, Youjiang Medical University for Nationalities, Baise, China; ^2^ Industrial College of Biomedicine and Health Industry, Youjiang Medical University for Nationalities, Baise, China; ^3^ Institute of Oceanographic Instrumentation, Qilu University of Technology (Shandong Academy of Sciences), Qingdao, China; ^4^ Key Laboratory of Advanced Energy Materials Chemistry (Ministry of Education), College of Chemistry, Nankai University, Tianjin, China; ^5^ School of Chemistry and Pharmaceutical Sciences, Guangxi Normal University, Guilin, China

**Keywords:** metal-organic frameworks, two-dimensional lamellar networks, oxygen evolution reaction, electrochemical activity, photocatalysis dye degradation

## Abstract

Two-dimensional (2D) metal-organic frameworks (MOFs) have been extensively investigated as oxygen evolution reaction (OER) materials because of their numerous advantages such as large specific surface areas, ultrathin thicknesses, well-defined active metal centers, and adjustable pore structures. Five Co-metal-organic frameworks, namely, [Co(L) (4.4′-bbidpe)H_2_O]_n_ [YMUN 1 (YMUN for Youjiang Medical University for Nationalities)], {[Co_2_(L)_2_ (4.4′-bbibp)_2_]·[Co^3^(L) (4.4′-bbibp)]·DMAC}_n_ (YMUN 2), [Co(L) (3,5-bip)]_n_ (YMUN 3), [Co(L) (1,4-bimb)]_n_ (YMUN 4), and [Co(L) (4.4′-bidpe)H_2_O]_n_ (YMUN 5), were designed and fabricated from flexible dicarboxylic acid 1,3-bis(4′-carboxylphenoxy)benzene (H_2_L) and rigid/flexible imidazole ligands. Their frameworks consist of two-dimensional lamellar networks with a number of differences in their details. Their frameworks are discussed and compared, and their oxygen evolution reaction electrochemical activities and photocatalysis dye degradation properties are investigated.

## Introduction

The development of green, clean, sustainable, and renewable energy storage and conversion technologies could effectively reduce the consumption of traditional fossil energies, mitigating deteriorating global environmental issues and achieving carbon-neutrality (Dinh et al., 2018; Roy et al., 2018; Li et al., 2019; Tian et al., 2019; Liang et al., 2020; Pan et al., 2021; Xue et al., 2021; Chang et al., 2022). Because of their high efficiencies, reliabilities, and environmental friendliness, water splitting, fuel cells, and metal-air batteries are the most promising of these technologies (Qin et al., 2020; Li C. et al., 2021; Du et al., 2021; Hu et al., 2021; Lu et al., 2021; Song et al., 2021; Zou et al., 2021). An electrochemical oxidation reaction, namely, the oxygen evolution reaction (OER), is the basic and crucial half-reaction that occurs in the overall processes of the abovementioned technologies (Li J. et al., 2021). It is well known that the high reaction energy barrier of this half-reaction is the main obstacle to practical applications of these technologies. Recently, noble metal-based materials (RuO_2_ and IrO_2_ for the OER) have been used and are considered state-of-the-art electrocatalysts. However, their low abundances in nature, high costs, and bonding instabilities hinder their practical applications (Li and Guo, 2019; Tripathy et al., 2019; Nemiwal et al., 2021; Yang et al., 2021). Therefore, the development of low-cost and high-efficiency OER electrocatalysts has become a major challenge for researchers in recent years.

Recently, non-noble metal (Fe, Co, Ni, Mn, and Cu) materials have garnered considerable interest as alternative electrocatalysts to noble metal-based materials due to their abundant reserves, low costs, and high catalytic activities (Wei et al., 2019; Wu et al., 2019; Sanad et al., 2021; Wu et al., 2021; Zhang et al., 2021). MOFs, as classical porous materials, are widely applied in heterogeneous catalysis, photocatalysis, gas separation, and sensing (Shi et al., 2019; Liu et al., 2020; Wang et al., 2020; Lu et al., 2022). Non-noble MOFs are an emerging class of OER electrocatalysts, and have shown great potential due to their large specific surface areas, abundant and tunable pore structures, structural diversities, high design flexibilities, and high porosities. The rational design and fabrication of MOF electrocatalysts with abundant and exposed active sites, enriched and accessible surface areas, and diversiform constructs allowing enhanced properties such as the electrical conductivity, electrochemical activity, and stability requires more attention if they are to be utilized in practical applications. The unique structures, diversiform porosities, and enriched specific surface areas of MOFs are conducive to their improved catalytic performances. Moreover, the active sites, pore structures, sizes, and morphologies of MOF materials can be accurately tuned throughout their entire structure. The development of advanced OER electrocatalysts is of great significance for the improvement of metal-air batteries, fuel cells, and water splitting technologies (Xie et al., 2019; Liang J. et al., 2021; Chen Y. et al., 2021; Liang Z. et al., 2021; Lourenco et al., 2021). Hierarchical 2D MOFs are excellent candidate electrocatalyst materials for improved OER performance. Compared with pure carbon-based OER catalysts, the simple synthetic methodologies, one-step reaction, and moderate reaction conditions for 2D MOFs make them easily obtainable, reducing both the synthetic complexity and the high-temperature processing progress. In addition, the uniform distribution of dense metal nodes generates a large number of accessible active sites on 2D MOFs, which is undoubtedly beneficial for improving their catalyst activities. The tunable compositions and controllable topologies of 2D MOFs, particularly with regard to mixed organic linkers and polymetallic nodes, are beneficial for regulating their electronic structure and the adsorption kinetics of active sites, thus improving their intrinsic activities. Therefore, the development of 2D MOF electrocatalyst materials is essential for fundamental research on the OER.

**SCHEME 1:**
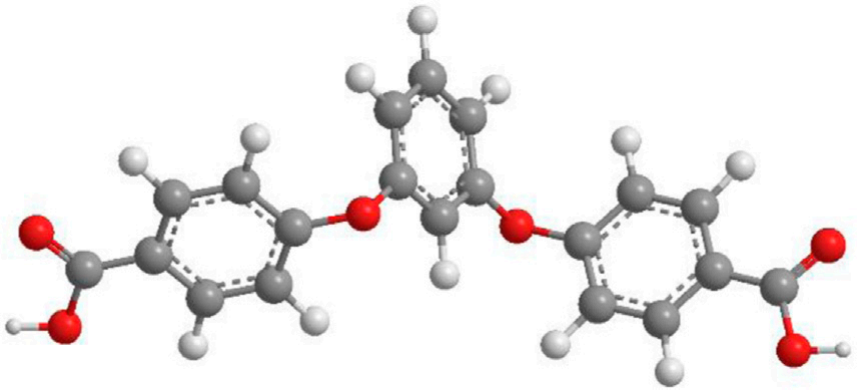
The selected O-donor organic ligand in YMUN 1–5 (the gray ball represents carbon, the white ball hydrogen, and the red ball oxygen).

**SCHEME 2:**
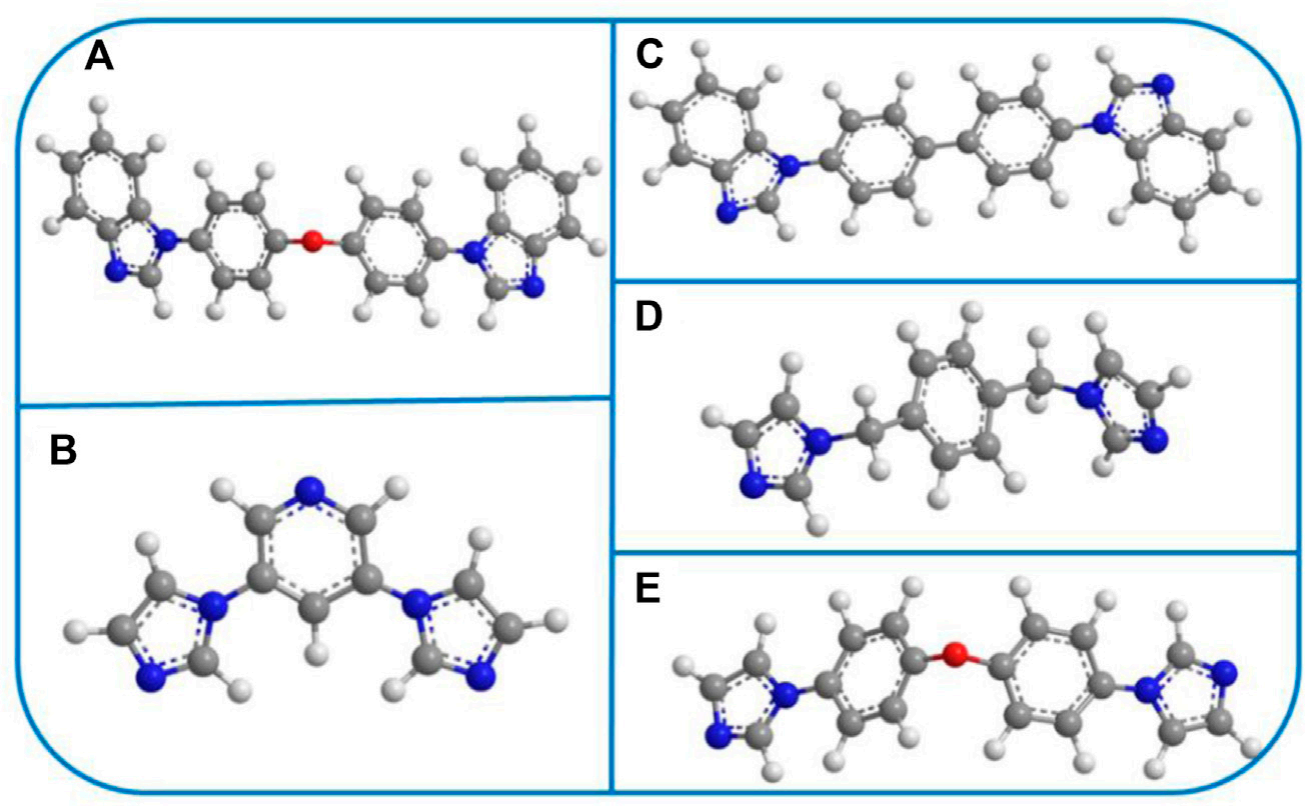
**(A–E)** The selected N-donor organic ligands in YMUN 1–5 (the gray ball represents carbon, the white ball hydrogen, the red ball oxygen, and the blue ball nitrogen).

2D MOFs present numerous advantages such as sheet-like structures and edges as well as unsaturated coordinative metal sites on their surfaces, which reduce their electrical resistance and increase electron transport, allowing superior OER electrical activity compared with 3D bulk MOFs. Therefore, in this work, a series of Co-MOFs, namely, [Co(L) (4.4′-bbidpe)H_2_O]_n_ (YMUN 1), {[Co_2_(L)_2_ (4.4′-bbibp)_2_]·[Co^3^(L) (4.4′-bbibp)]·DMAC}_n_ (YMUN 2), [Co(L) (3,5-bip)]_n_ (YMUN 3), [Co(L) (1,4-bimb)]_n_ (YMUN 4), and [Co(L) (4.4′-bidpe)H_2_O]_n_ (YMUN 5) were designed and synthesized from flexible 1,3-bis(4′-carboxylphenoxy)benzene (H_2_L, Scheme 1) and various imidazole ligands (Scheme 2) using solvothermal methods. The Co-MOFs exhibited different 2D lamellar networks; the frameworks of 1–5 were discussed and compared. Their OER electrochemical activities and photocatalysis dye degradation properties were investigated.

## Experimental section

### Materials and general characteristics

Please refer to the Supplementary Material for information on the materials used and the general characteristics of the MOFs.

### Synthesis of Co-metal-organic frameworks

The five MOFs (YMUN 1–5) were obtained in crystalline form using a solvothermal method. A Teflon-lined stainless-steel autoclave (23 ml) was used to synthesize YMUN 1. The reagents were mixed under stirring for 0.5 h, transferred to the autoclave, and heated to 130 C for 72 h (heating rate: 1 min/°C from 30 to 130 C), followed by cooling to ambient temperature (rate: 5 min/°C). Violet, blocky crystals were obtained. The syntheses of 2–5 were similar to that of one and all obtained products were identical in color.

[Co(L) (4.4′-bbidpe)H_2_O]_n_ (YMUN 1).

H_2_L (0.035 g, 0.1 mmol), 4,4′-bbidpe (0.080 g, 0.2 mmol), Co(NO_3_)_2_·6H_2_O (0.058 g, 0.20 mmol), DI water, and N,N-dimethylacetamide (DMAC) were used as reagents. Elemental analysis (%): calculated for C_46_H_32_CoN_4_O_8_: C, 66.75; H, 3.90; N, 6.77. Found: C, 66.25; H, 3.40; N, 6.07. Yield: 45 mg, 0.054 mmol, and 54% based on H_2_L. IR (KBr, cm^−1^): 3,631 (m), 3,436 (w), 1920 (w), 1,595 (s), 1,544 (m), 1,507 (s), 1,479 (s), 1,462 (m), 1,419 (s), 1,396 (s), 1,323 (w), 1,301 (m), 1,269 (s), 1,229 (s), 1,161 (s), 1,122 (m), 1,096 (w), 1,011 (w), 990 (m), 969 (m), 909 (w), 870 (m), 832 (m), 781 (m), 742 (m), 696 (w), 651 (w), 617 (w), 576 (w), 532 (w), 464 (w), and 430 (w).

{[Co_2_(L)_2_ (4.4′-bbibp)_2_]·[Co^3^(L) (4.4′-bbibp)]·DMAC}_n_ (YMUN 2).

H_2_L (0.035 g, 0.1 mmol), 4,4′-bbibp (0.079 g, 0.2 mmol), Co(NO_3_)_2_·6H_2_O (0.058 g, 0.20 mmol), DI water, and N,N- -dimethylacetamide (DMAC) were used as reagents. Elemental analysis (%): calculated for C_142_H_101_Co_3_N_13_O_20_: C, 68.60; H, 4.10; N, 7.32. Found: C, 67.85; H, 3.90; N, 6.70. Yield: 49 mg, 0.02 mmol, and 59% based on H_2_L. IR (KBr, cm^−1^): 3,417 (w), 1,626 (m), 1,591 (w), 1,566 (w), 1,508 (s), 1,479 (m), 1,458 (m), 1,405 (s), 1,297 (w), 1,264 (m), 1,223 (s), 1,165 (m), 1,144 (w), 1,229 (s), 1,119 (w), 1,012 (w), 966 (w), 858 (w), 830 (w), 818 (w), 784 (w), 735 (w), 710 (w), 646 (w), 616 (w), 584 (w), 531 (w), and 461 (w).

[Co(L) (3,5-bip)]_n_ (YMUN 3).

H_2_L (0.035 g, 0.1 mmol), 3,5-bip (0.046 g, 0.2 mmol), Co(NO_3_)_2_·6H_2_O (0.058 g, 0.20 mmol), DI water, and N,N-dimethylformamide (DMF) were used as reagents. Elemental analysis (%): calculated for C_31_H_23_CoN_5_O_7_: C, 58.50; H, 3.64; N, 11.00. Found: C, 58.10; H, 3.10; N, 10.20. Yield: 41 mg, 0.065 mmol, and 65% based on H_2_L. IR (KBr, cm^−1^): 3,436 (m), 3,143 (w), 1,599 (s), 1,561 (m), 1,507 (s), 1,479 (m), 1,416 (w), 1,378 (s), 1,312 (w), 1,266 (m), 1,226 (s), 1,165 (m), 1,123 (w), 1,068 (w), 1,012 (w), 969 (w), 862 (w), 832 (w), 784 (m), 744 (w), 696 (w), 651 (w), 497 (w), and 467 (w).

[Co(L) (1,4-bimb)]_n_ (YMUN 4).

H_2_L (0.035 g, 0.1 mmol), 1,4-bimb (0.048 g, 0.2 mmol), Co(NO_3_)_2_·6H_2_O (0.058 g, 0.20 mmol), DI water, and N,N-dimethylacetamide (DMAC) were used as reagents. Elemental analysis (%): calculated for C_34_H_26_CoN_4_O_6_: C, 63.26; H, 4.06; N, 8.68. Found: C, 62.80; H, 3.90; N, 8.10. Yield: 46 mg, 0.072 mmol, and 72% based on H_2_L. IR (KBr, cm^−1^): 3,135 (m), 1,591 (s), 1,562 (w), 1,520 (m), 1,499 (w), 1,478 (s), 1,442 (m), 1,411 (s), 1,388 (s), 1,263 (m), 1,218 (s), 1,158 (m), 1,110 (s), 1,091 (m), 1,013 (w), 963 (s), 946 (m), 856 (m), 827 (w), 795 (w), 783 (w), 771 (w), 751 (m), 687 (w), 655 (m), 620 (w), 594 (w), 493 (w), and 474 (w).

[Co(L) (4.4′-bidpe)H_2_O]_n_ (YMUN 5).

H_2_L (0.035 g, 0.1 mmol), 4,4′-bidpe (0.060 g, 0.2 mmol), Co(NO_3_)_2_·6H_2_O (0.058 g, 0.20 mmol), DI water, and N,N-dimethylacetamide (DMAC) were used as reagents. Elemental analysis (%): calculated for C_38_H_28_CoN_4_O_8_: C, 62.73; H, 3.88; N, 7.70. Found: C, 62.10; H, 3.20; N, 6.90. Yield: 44 mg, 0.061 mmol, and 61% based on H_2_L. IR (KBr, cm^−1^): 3,446 (m), 3,386 (w), 1,594 (s), 1,514 (s), 1,480 (s), 1,397 (s), 1,304 (w), 1,235 (s), 1,214 (s), 1,160 (m), 1,120 (m), 1,063 (m), 1,011 (w), 966 (s), 855 (m), 830 (m), 800 (m), 780 (m), 740 (m), 705 (w), 654 (m), 616 (w), 552 (w), 526 (w), and 492 (w).

The crystallographic data and structure refinement for YMUN 1–5 are summarized in Table 1. Supplementary Table S1 lists the related bond lengths and angles in 1–5. The CCDC numbers for 1–5 are 2195002, 2195003, 2194999, 2195001, and 2195000, respectively.

**TABLE 1:** Summary of crystal data and structure refinement parameters for YMUN 1–5^a^.

Empirical formula	C_46_H_32_CoN_4_O_8_	C_142_H_101_Co_3_N_13_O_20_	C_31_H_21_CoN_5_O_6_·H_2_O	C_34_H_26_CoN_4_O_6_	C_38_H_28_CoN_4_O_8_
CCDC. NO.	2195002	2195003	2194999	2195001	2195000
Formula weight	827.68	2,486.14	636.47	645.52	727.57
Crystal system	Monoclinic	Monoclinic	Triclinic	Monoclinic	Monoclinic
Space group	*P*2_1_/*n*	*P*2_1_/*n*	*P*ī	*P*2_1_/*n*	*P*2_1_/*n*
*a* (Å)	16.9254 (4)	15.8024 (3)	8.9666 (3)	7.3088 (9)	15.4621 (12)
*b* (Å)	12.2504 (4)	42.0723 (11)	11.8244 (3)	24.077 (4)	11.9109 (9)
*c* (Å)	19.3853 (5)	19.6622 (4)	15.0313 (4)	16.768 (2)	17.5891 (16)
*α* (°)	90	90	68.051 (1)	90	90
*β* (°)	110.471 (1)	107.605 (1)	86.439 (1)	92.819 (4)	102.271 (3)
*γ* (°)	90	90	71.615 (1)	90	90
*V* (Å^3^)	3,765.57 (18)	12,460.0 (5)	1,399.91 (7)	2,947.1 (7)	3,165.3 (4)
*Z*	4	4	2	4	4
*D*_calcd_ (Mg∙m^−3^)	1.460	1.325	1.510	1.455	1.513
*µ* (mm^−1^)	0.52	0.47	0.67	0.64	0.61
Reflections collected	5,491	14,952	4,703	3,556	4,174
Data/parameters	7,677/539	25,445/1,609	5,693/400	5,872/406	6,451/461
*F* (000)	1708	5,140	654	1,332	1,500
*T* (K)	170	170	170	170	150
*R*_int_	0.057	0.079	0.035	0.101	0.063
Final R indices [*I* > 2sigma(I)]	*R*_1_ = 0.046 *wR*_2_ = 0.106	*R*_1_ = 0.0560 *wR*_2_ = 0.1409	*R*_1_ = 0.0355 *wR*_2_ = 0.0821	*R*_1_ = 0.0728 *wR*_2_ = 0.1640	*R*_1_ = 0.0614 *wR*_2_ = 0.186
R indices (all data)	*R*_1_ = 0.0792 *wR*_2_ = 0.0891<	*R*_1_ = 0.1180 *wR*_2_ = 0.1119	*R*_1_ = 0.0509 *wR*_2_ = 0.0695	*R*_1_ = 0.1366 *wR*_2_ = 0.1391	*R*_1_ = 0.1088 *wR*_2_ = 0.1864<
Gof	1.06	1.01	1.08		1.06

^a^ *R*_1_ = Σǁ*F*_*o*_| − |*F*_*c*_ǁ/Σ|*F*_*o*_|, _*w*_ *R*_2_ = [Σ_w_ (*F*_*o*_^2^− *F*_*c*_^2^)^2^]/[*Σ*_*w*_ (*F*_*o*_^2^)^2^]^1/2^.

## Results and discussion

### Structure analysis

[Co(L) (4.4′-bbidpe)H_2_O]_n_ (YMUN 1).

**FIGURE 1:**
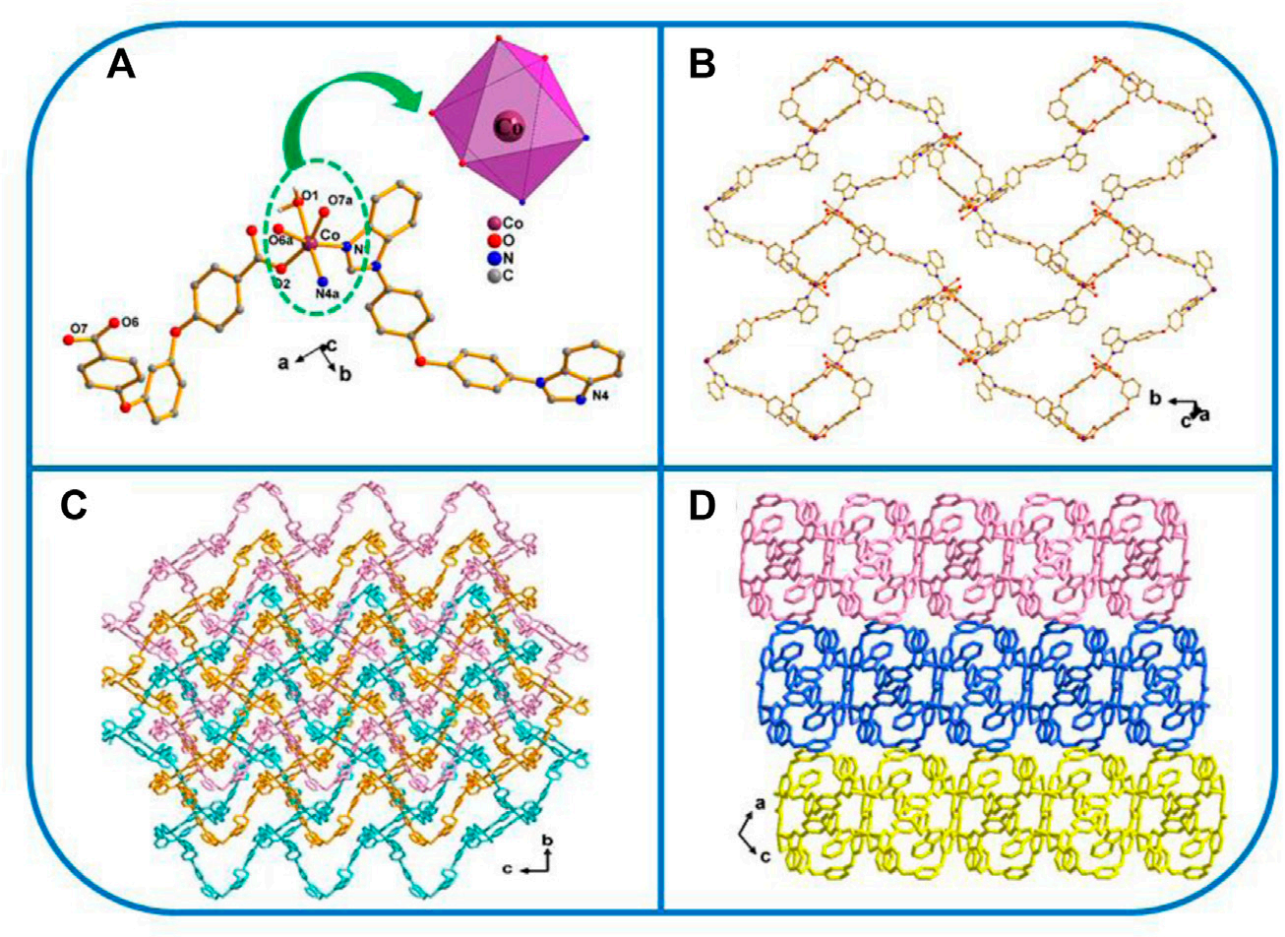
**(A)** Coordination unit of the Co ion in YMUN 1 ((hydrogen atoms are omitted for clarity), symmetry codes: (i) -*x*+2, -*y*, -*z*+1; (ii) *x*+1/2, -*y*+3/2, *z*+1/2; (iii) *x*-1/2, -*y*+3/2, *z*-1/2.); **(B)** view of the single 2D lamellar structure of 1; **(C)** view of the three-fold interpenetrating 2D stratified network through the 2D lamellar structure of one along the *a* axis; and **(D)** view of the 3D framework through the 2D stratified network with weak interactions occurring along the *b* axis.

**SCHEME 3:**
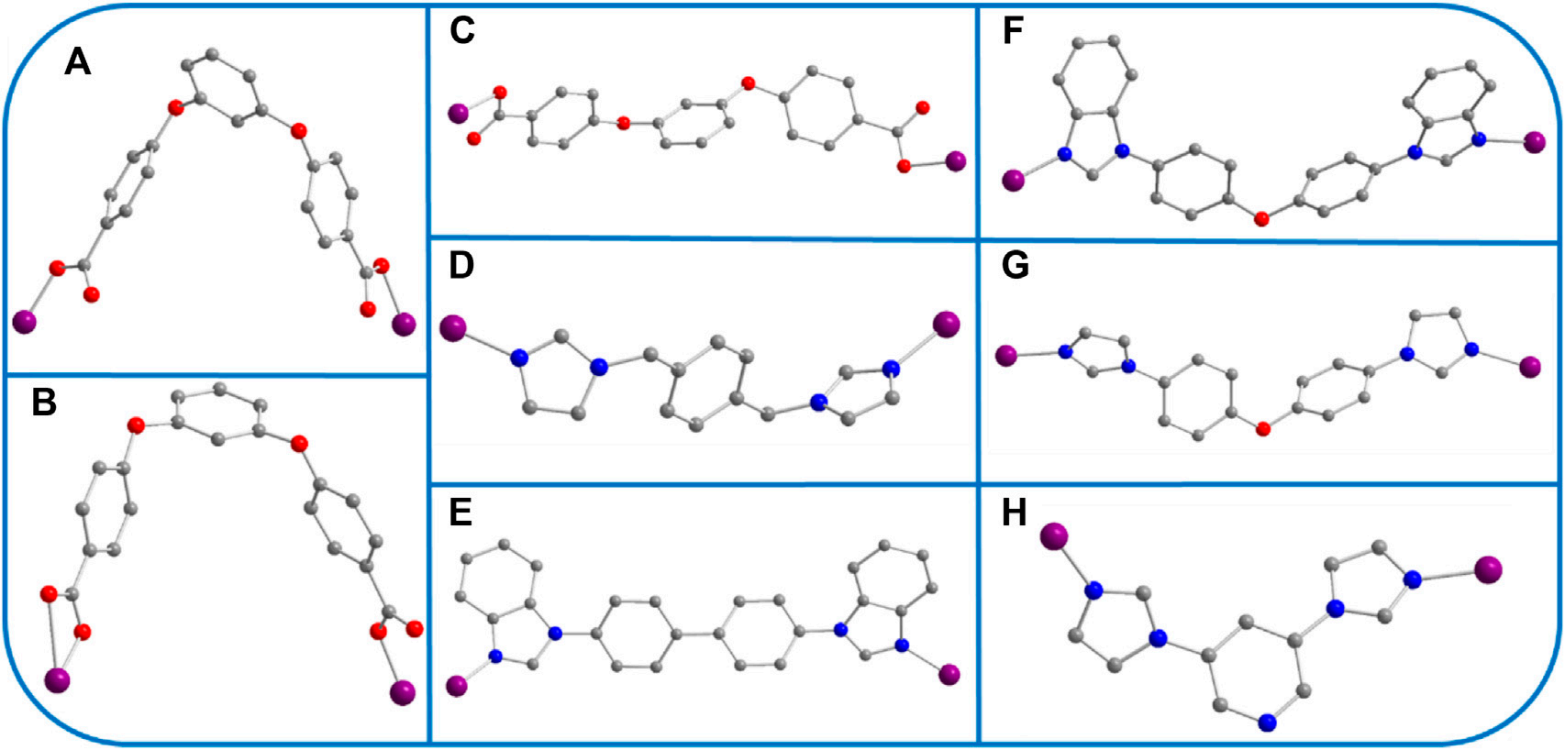
**(A–H)** The various coordination modes of O/N-donor ligands in YMUN 1–5 (the gray ball represents carbon, the red ball oxygen, the blue ball nitrogen, the violet ball cobalt, and hydrogen atoms are omitted for clarity).

One exhibits the *P*2_1_/*n* space group of the monoclinic system, determined by crystal data analysis. The asymmetric unit comprises one Co atom, one coordinated water molecule, 1 L^2−^ ligand, and one 4,4′-bbidpe ligand (Figure 1A). The H_2_L carboxylate ligand presents a *µ*_2_-η^1^:η^1^:η^1^:η^0^ (L^2−^, Scheme 3B) coordinated mode while that of the imidazole ligand (4.4′-bbidpe) is *µ*_2_-η^1^:η^1^ (Scheme 3F). The Co atom is six-fold coordinated by four oxygen atoms from one water molecule and from the two–COO^−^ groups in 2 L^2−^ ligands, and by two nitrogen atoms from two isolated 4,4′-bbidpe imidazole ligands, giving a distorted octahedron (CoO_4_N_2_) coordination geometry. The neighboring L^2−^ and 4,4′-bbidpe ligands connect, forming a 2D lamellar structure (Figure 1B). Three neighbor 2D lamellar structures interact with one another, forming a 2D three-fold interpenetrating stratified network, based on the spacious nature of a single 2D lamellar structure (Figure 1C). Finally, these neighboring interpenetrating 2D stratified networks are balanced to form a 3D framework through weak π···π interactions (Figure 1D).

{[Co_2_(L)_2_ (4.4′-bbibp)_2_]·[Co^3^(L) (4.4′-bbibp)]·DMAC}_n_ (YMUN 2).

**FIGURE 2:**
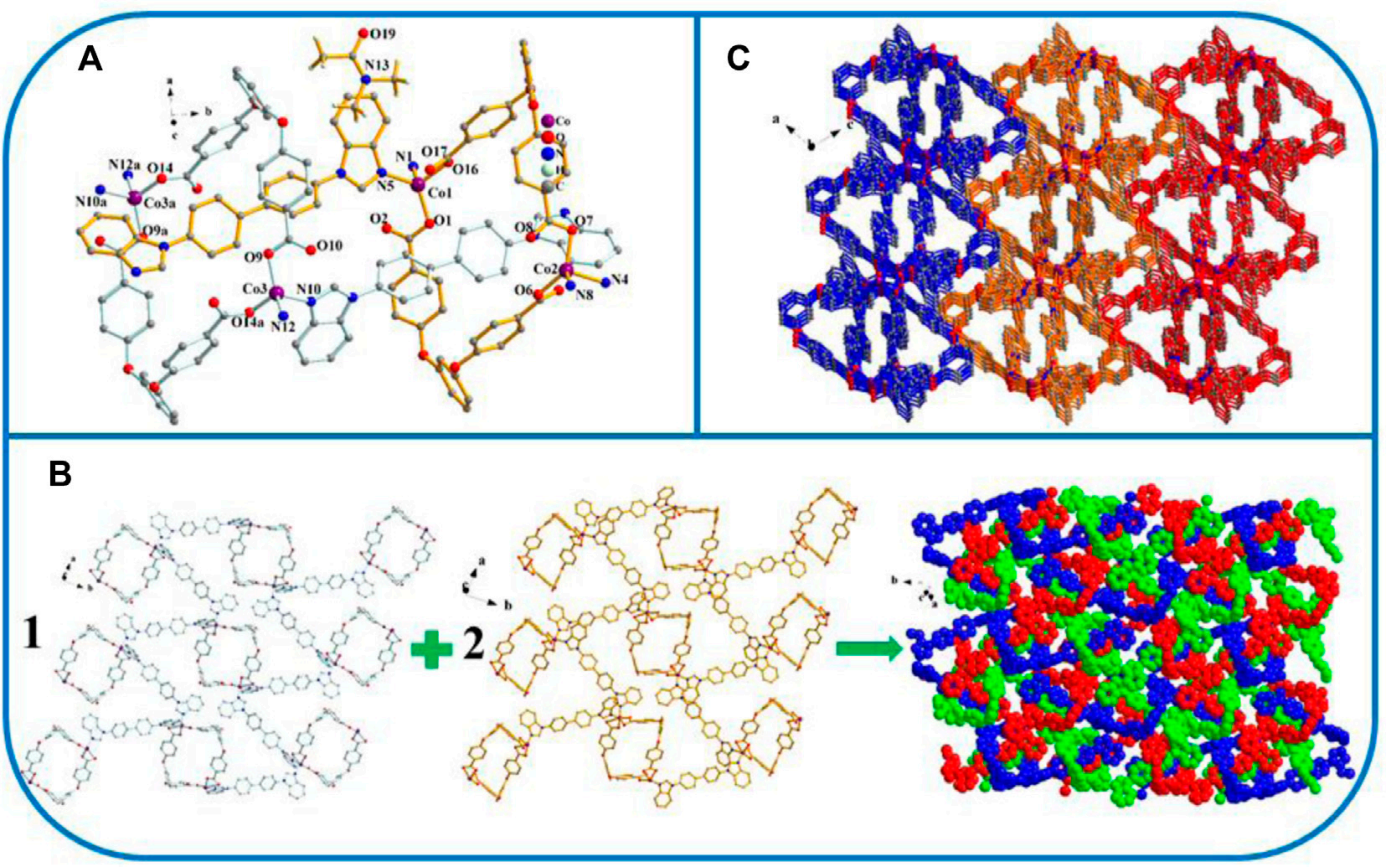
**(A)** Coordination environment of the Co ion in YMUN 2 ((hydrogen atoms are omitted for clarity), symmetry codes: (i) -*x*+5/2, *y*+1/2, -*z*+5/2; (ii) -*x*+3/2, *y*+1/2, -*z*+3/2; (iii) -*x*+1, -*y*+2, -*z*+1; (iv) *x*-1/2, -*y*+3/2, *z*-1/2; (v) -*x*+5/2, *y*-1/2, -*z*+5/2; (vi) -*x*+3/2, *y*-1/2, -*z*+3/2; (vii) *x*+1/2, -*y*+3/2, *z*+1/2.); **(B)** view of the three-fold interpenetrating 2D stratified network through the different 2D lamellar structures of 2; **(C)** view of the 3D framework through the 2D stratified network with weak interactions occurring along the *b* axis.

The crystal data reveal that two exhibits the *P*2_1_/*n* space group of the monoclinic system. Its asymmetric unit consists of three Co atoms, 3 L^2−^ ligands, three 4,4′-bbibp ligands, and one DMAC molecule (Figure 2A). As shown in Scheme 3, the coordinated mode of the H_2_L carboxylate ligand is *µ*_2_-η^1^:η^0^:η^1^:η^0^ (L^2−^, Scheme 3A) while that of the imidazole ligand (4.4′-bbibp) is *µ*_2_-η^1^:η^1^ (Scheme 3E). The coordination environments of Co^1^, Co^2^, and Co^3^ are identical; four-fold coordinated by two oxygen atoms from the two–COO^−^ groups in two HL^4−^ ligands, and by two nitrogen atoms from two independent 4,4′-bbibp imidazole ligands. The three Co ions differ in the bond lengths and angles of Co–O/N and the bonding angle of O/N-donor ligands. Based on these discrepancies, the neighboring L^2−^ and 4,4′-bbidpe ligands connect through Co ion metal centers, forming two different kinds of 2D lamellar structures. The compositional ratio of the two different 2D lamellar structures in the three-fold interpenetrating 2D stratified network is 1:2 (Figure 2B). Finally, the neighboring interpenetrating 2D stratified networks are balanced to form a 3D framework through weak π···π interactions (Figure 2C).

[Co(L) (3,5-bip)]_n_ (YMUN 3).

**FIGURE 3:**
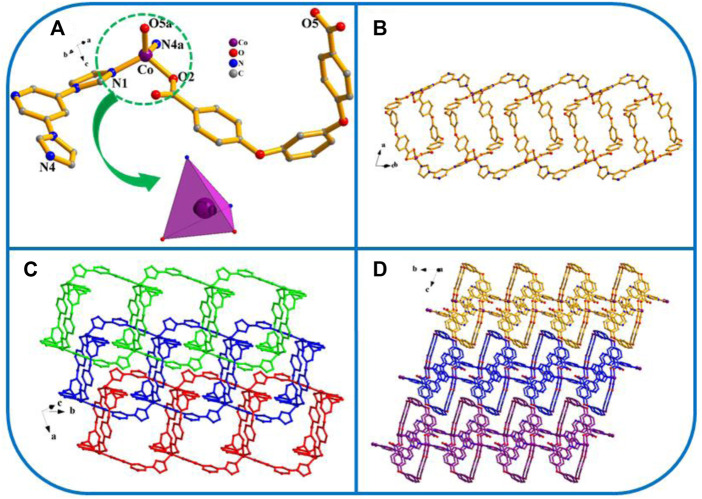
**(A)** Coordination unit of the Co ion in YMUN 3 ((hydrogen atoms are omitted for clarity), symmetry codes: (i) -*x*+1, -*y*, -*z*+1; (ii) *x*, *y*-1, *z*; (iii) *x*, *y*+1, *z*.); **(B)** view of the single 1D structure of 3; **(C)** view of the mutually-interpenetrating 2D network through the identical 1D structures of 3; **(D)** view of the 3D framework through the 2D stratified network with weak interactions occurring along the *a* axis.

Three exhibits the *P*ī space group of the triclinic system, determined by crystal data analysis. The asymmetric unit comprises one Co atom, one 3,5-bip ligand, and 1 L^2−^ ligand (Figure 3A). As shown in Scheme 3, the H_2_L carboxylate ligand presents a *µ*_2_-η^1^:η^0^:η^1^:η^0^ (L^2−^, Scheme 3A) coordinated mode while that of the imidazole ligand (3,5-bip) is *µ*_2_-η^1^:η^1^ (Scheme 3H). The Co atom is four-fold coordinated by two oxygen atoms from 2 L^2−^ ligands, and by two nitrogen atoms from two isolated 3,5-bip imidazole ligands, giving a distorted tetrahedron (CoO_2_N_2_) coordination geometry. The H_2_L carboxylate ligands connect through the Co ions, forming one toroidal structure; these toroidal structures then connect with the 3,5-bip imidazole ligands to form a 1D container-like structure (Figure 3B). As shown in Figure 3C, the 1D container-like structures then interlock with one another to form interpenetrating 2D polymer networks. Finally, the interpenetrating 2D polymer networks are balanced to form a 3D framework through weak π···π interactions (Figure 3D).

[Co(L) (1,4-bimb)]_n_ (YMUN 4).

**FIGURE 4:**
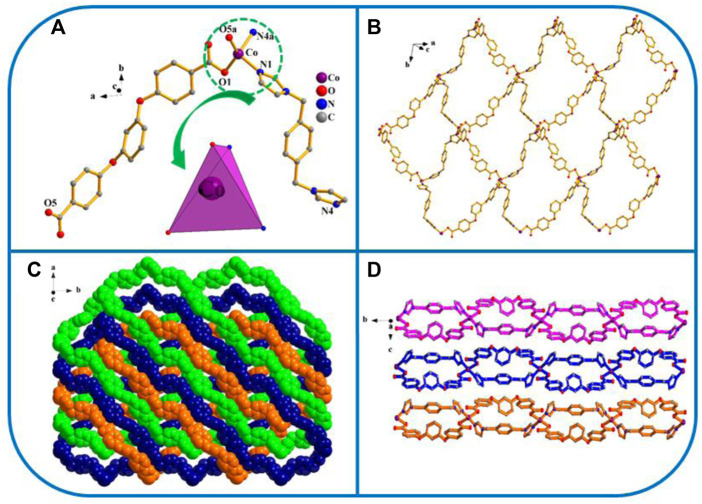
**(A)** Coordination unit of the Co ion in YMUN 4 ((hydrogen atoms are omitted for clarity), symmetry codes: (i) -*x*, *y*+1/2, -*z*+3/2; (ii) -*x*+3, *y*+1/2, -*z*+3/2; (iii) -*x*, *y*-1/2, -*z*+3/2; (iv) -*x*+3, *y*-1/2, -*z*+3/2.); **(B)** view of the single 2D lamellar structure of 4; **(C)** view of the three-fold interpenetrating 2D stratified network through the identical 2D lamellar structures of four along the *c* axis; **(D)** view of the 3D framework through the 2D stratified network with weak interactions occurring along the *a* axis.

Four exhibits the *P*2_1_/*n* space group of the monoclinic system, determined by crystal data analysis. The asymmetric unit comprises one Co atom, 1 L^2−^ ligand, and one 1,4-bimb ligand (Figure 4A). As shown in Scheme 3, the H_2_L carboxylate ligand presents a *µ*_2_-η^1^:η^0^:η^1^:η^0^ (L^2−^, Scheme 3C) coordinated mode while that of the imidazole ligand (1,4-bimb) is *µ*_2_-η^1^:η^1^ (Scheme 3D). As shown in Figure 4, the final framework of four is similar to that of 2.

[Co(L) (4.4′-bidpe)H_2_O]_n_ (YMUN 5).

**FIGURE 5:**
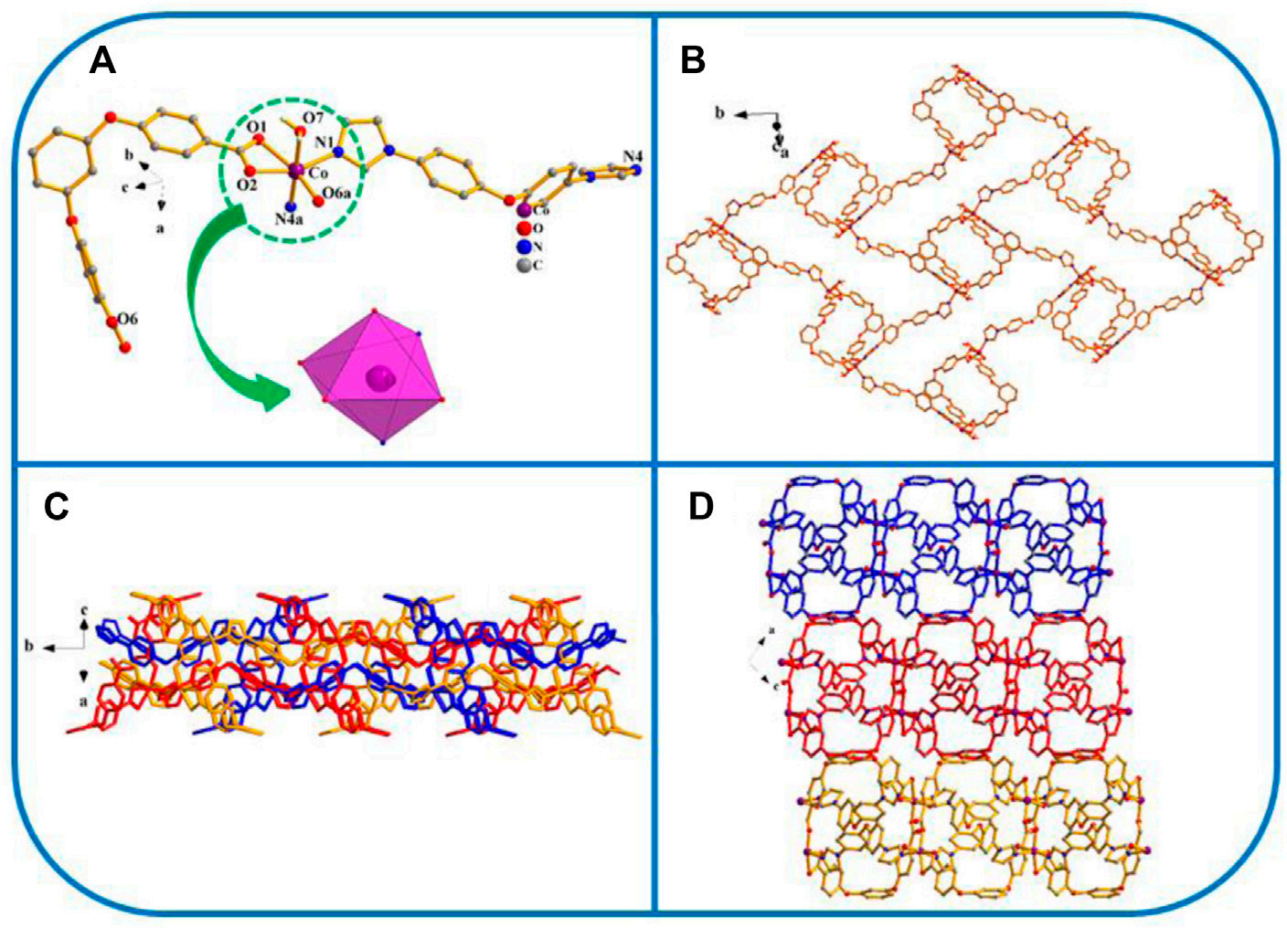
**(A)** Coordination unit of the Co ion in YMUN 5 ((hydrogen atoms are omitted for clarity), symmetry codes: (i) -*x*+1, -*y*, -*z*+1; (ii) *x*-1/2, -*y*+3/2, *z*-1/2; (iii) *x*+1/2, -*y*+3/2, *z*+1/2.); **(B)** view of the single 2D lamellar structure of 5; **(C)** view of the three-fold interpenetrating 2D stratified network through the identical 2D lamellar structures of 5; **(D)** view of the 3D framework through the 2D stratified network with weak interactions occurring along the *b* axis.

Fiveexhibits the *P*2_1_/*n* space group of the monoclinic system, determined by crystal data analysis. As shown in Figure 5, the coordination mode, formation process, and final structure of five are similar to those of 1.

### Structural discussion and comparison

In this work, a flexible H_2_L ligand, acting as a main ligand, was used to synthesize five types of metal organic framework materials containing cobalt ions and different imidazole ligands. As shown in Scheme 3, three coordination modes existed for the flexible H_2_L ligand in YMUN 1–5, owing to the different auxiliary ligands (flexible 4,4′-bbidpe, rigid 4,4′-bbibp, rigid 3,5-bip, flexible 1,4-bimb, and flexible 4,4′-bidpe) and reaction solvents used. The interpenetrated frameworks were formed based on the flexibility and various configurations of the main H_2_L ligand. The O/N-donor ligands connected with one another to form 2D layered structures in 1, 2, 4, and 5, and a 1D chain in three due to the length and flexibility of the auxiliary ligands. Finally, the formation of three-fold interpenetrating 2D stratified networks is different for 1, 2, 4, and five compared with 3. The six imidazole auxiliary ligands all exhibit syn conformations (Schemes 3D–H). The frameworks were obtained using two different types of solvent reactions (DI water and DMAC (8 ml, v/v = 1:1), 130 C for 1, 2, 4, and 5, and DI water and DMF (8 ml, v/v = 1:1), 130 C for 3). Thus, a series of Co MOFs were successfully designed by utilizing different imidazole auxiliary ligands and reaction solvents.

### Thermal stability and purity

The thermogravimetric stabilities of YMUN 1–5, which are vital evaluation parameters of their potential performance in catalytic applications, were determined. The TG curves, as displayed in Supplementary Figure S1, reveal that the entire skeleton structures of 1–5 remained intact up to 300 C. The skeletons of 1–5 collapsed at temperatures of 320 C, 355 C, 322 C, 360 C, and 348 C, respectively. PXRD measurements were performed to evaluate the phase purities of 1–5. The PXRD profiles show that the characteristic diffraction peaks obtained for 1–5 match well with those obtained for simulated single crystal PXRD patterns (Supplementary Figures S2–S6). The preeminent thermal stabilities and phase purities of 1–5 indicate that they should perform exceptionally in practical applications.

### Microstructure and porosity analysis

The specific surface area and porosity are prominent characteristics of 2D MOF electrocatalysis materials. The morphologies of two and four were studied using SEM (Supplementary Figure S7). These MOFs display a bulk structure composed of numerous stacked sheet-like layers, which favor the transport of electrolytes and gas emissions. BET gas-sorption measurements demonstrate the porosities of the 2D MOFs. According to IUPAC classification, the adsorption performance of two and four corresponds to type-III isotherms. The BET specific surface areas of two and four are approximately 1.50 m^2^ g^−1^ and 1.55 m^2^ g^−1^, respectively, and the average pore diameters are approximately 18.7 nm and 14.6 nm, respectively, owing to the presence of flaky cracks in the composites. (Supplementary Figure S8, Supplementary Table S2). These results demonstrate that two and four exhibit a mesoporous structure, providing enough space for internal active sites and allowing them to be fully exposed.

### Electrochemical evaluation of YMUN 1–5 for the oxygen evolution reaction

**FIGURE 6:**
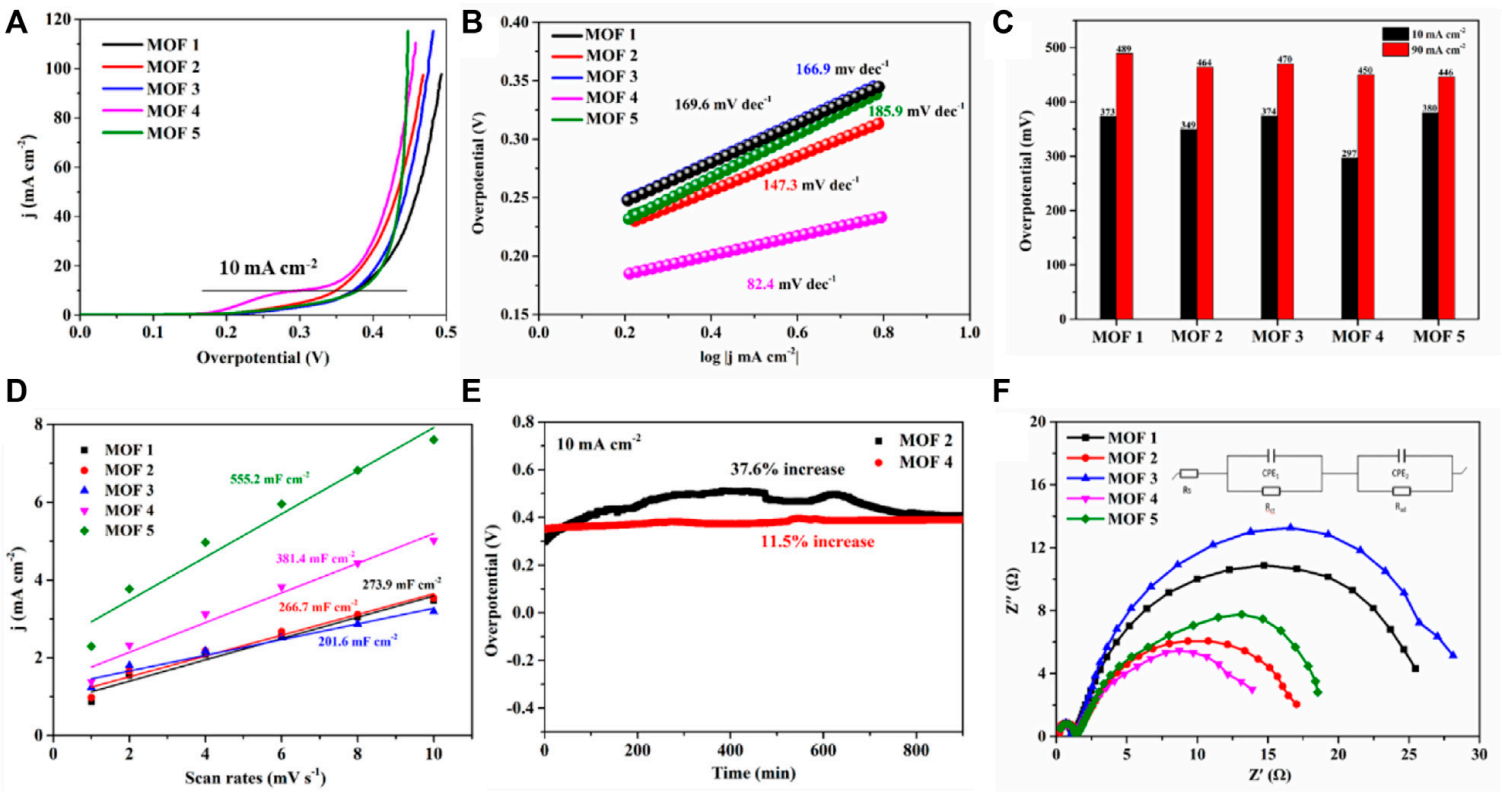
**(A)** LSV curves; **(B)** Tafel plots; **(C)** summary of overpotentials (at 10 and 90 mA cm^−2^); **(D)** double-layer capacitance (C_dl_) plots for YMUN 1–5; **(E)** stabilities of two and four for the OER; **(F)** Nyquist plots for 1–5 in a 1.0 M KOH solution.

The OER properties of 1–5 were investigated based on the extensive electrochemical applications of transition metal cobalt polymer materials (Chen C. et al., 2021; Liang et al., 2022; Peng et al., 2022). The electrocatalytic performances of 1–5 for the OER were examined in a 1.0 M alkaline KOH electrolyte at room temperature using a three-electrode setup. As shown in Figure 6A, the LSV curves of 1–5 show that they exhibited overpotentials of 373 mV for 1, 349 mV for 2, 374 mV for 3, 297 mV for 4, and 380 mV for 5, at a current density of 10 mA cm^−2^ and a scan rate of 5 mV s^−1^ (Table 2). The overpotential of four was lower than the other four catalyst materials. Notably, four also exhibited a lower overpotential of 450 mV at a higher current density of 90 mA cm^−2^ (Figure 6C). The OER reaction kinetics were investigated using the calculated Tafel plots obtained from linear sweep voltammetry (LSV) data. The Tafel slopes of 1–5 were 169.6 mV dec^−1^, 147.3 mV dec^−1^, 166.9 mV dec^−1^, 82.4 mV dec^−1^, 185.9 mV dec^−1^, respectively; the Tafel slope of four was lowest, illustrating favorable reaction dynamics which can significantly increase the OER rate and overpotentials (Figure 6B).

**TABLE 2:** Summarized OER catalytic parameters of different catalysts in a 1.0 M KOH electrolyte.

Catalyst	η [mV]	Tafel slope [mV·dec^−1^]	C_dl_ [mF·cm^−2^]	R_ct_ [Ω]
YMUN 1	373	169.6	52	1.10
YMUN 2	349	147.3	50	1.38
YMUN 3	374	166.9	38	1.05
YMUN 4	297	82.4	72	1.21
YMUN 5	380	185.9	105	1.25

To further understand the OER catalytic activity of 1–5, their electrochemically active surface areas (ECSA) were determined. The ESCAs were calculated from the electrochemical double-layer capacitances (C_dl_) obtained from cyclic voltammetry (CV) curves at varying scan rates (Supplementary Figures S9–S13). The following equation was utilized: ESCA = C_dl_/C_s_, where C_s_ is the capacitance per unit area of a smooth surface in an electrocatalyst material. An average value for C_s_ (40 μF cm^−2^) was used in this work since C_s_ is usually determined to be between 20 and 60 μF cm^−2^ in a 1.0 M KOH solution (Nai and Lou, 2019; Peng et al., 2022). As shown in Figure 6D, the C_dl_ values of 1–5 were 52 mF cm^−2^ for 1, 50 mF cm^−2^ for 2, 38 mF cm^−2^ for 3, 72 mF cm^−2^ for 4, and 105 mF cm^−2^ for 5. The ECSA value of four was 180 cm^−2^; higher than those of 1–3. The ECSA results show that 1–5 contain numerous active sites, which increase the possibility of contact between reactants and active components, hence accelerating the OER process (Nai and Lou, 2019).

Robust durability is an essential factor for outstanding electrochemical activity in practical applications. Two and four were selected to evaluate the OER long-term stability. Potential increases of approximately 37.6% for 2 and 11.5% for four were observed at a stabilized current density after 54,000 s of galvanostatic operation at 10 mA cm^−2^. To further determine the prominent OER performances of 1–5, electrochemical impedance spectroscopy (EIS) was performed to probe the transfer kinetics of charge carriers. Nyquist plots (Figure 6F) obtained from EIS measurements at 1.55 V (vs. RHE), displayed small electrolyte resistance values (R_s_ = 0.25 Ω, 0.23 Ω, 0.20 Ω, 0.22 Ω, and 0.23 Ω) for 1–5. The charge transfer resistances (R_ct_) of 1–5 were 1.10 Ω, 1.38 Ω, 1.05 Ω, 1.21Ω, and 1.25 Ω, respectively; well-correlated with the fast water oxidation kinetics, which were small and similar. Notably, two semicircles were observed in the Nyquist plots of 1–5, indicative of two time-constant behaviors (Figure 6F). The first behavior reflects the charge transfer resistance (R_ct_) that appeared in the high-frequency region. The observed low frequency semicircles correspond to the adsorption of reaction intermediates on the electrode surface, and represent the hydroxide transition properties on the surface of open metal sites (Gao et al., 2017; Jiang et al., 2017). The small values of R_c_ and R_ct_ resulted in a lower applied potential and overpotential.

Based on the above test analyses, 1–5 exhibited excellent OER performances; the catalytic activities of two and four were superior. The accessibility of active sites and the electrode geometry are often key factors affecting the electrocatalytic activity. As discussed in the structural analysis section, the metal centers in two and four are four-fold coordinated with two vacant coordination sites. The five frameworks are 2D layered, three-fold interpenetrating networks; however, the formation mechanisms were different. The mechanism for framework three is 1D + 1D → 2D with a three-fold interpenetrating network, whereas 1, 2, 4, and five are 2D + 2D → 2D with three-fold interpenetrating networks. The potential active sites in the 2D layered networks are more accessible for reactants. Thus, the advantageous, discretely- and homogeneously-distributed metal nodes in these MOFs offer exceptional platforms for efficient OER.

### Photocatalytic properties of YMUN 1–5 for organic dyes

**FIGURE 7:**
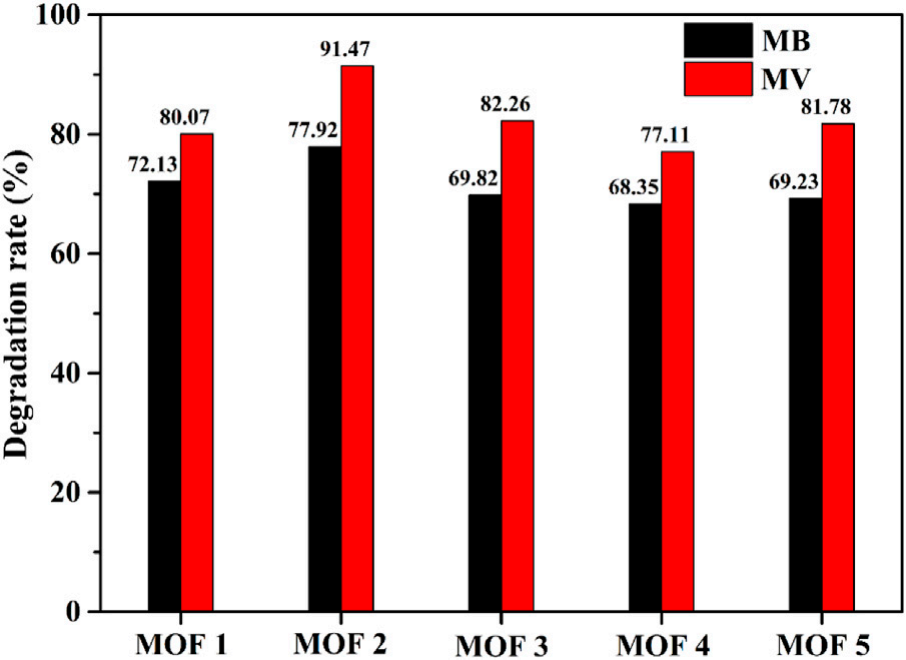
Degradation rates of MB/MV dye solutions with YMUN 1–5 under UV irradiation.

Photocatalysis could be an efficient way to degrade organic dyes for purifying wastewater. Cobalt organic framework photocatalysts are widely used to decompose organic dyes in water purification processes, owing to their highly active metal center (Fan et al., 2018). Therefore, 1–5 were employed to decompose the common organic dyes methyl violet (MV) and methylene blue (MB), under UV irradiation, in order to evaluate their photocatalytic efficiencies for wastewater purification. The degradation activities of 1–5 in MB/MV solutions were monitored over a time period of 0–180 min using spectroscopy (Supplementary Figures S14–S18). As shown in Figure 7, the degradation ratios of 1–5 for MB were 72.13%, 77.92%, 69.82%, 68.35%, and 69.23%, respectively. The degradation ratios of 1–5 for MV were 80.07%, 91.47%, 82.26%, 77.11%, and 81.78%, respectively. The catalytic activities of 1–5 were higher for MV than MB, and two exhibited the highest degradation ratio. Therefore, two could be used as a potential photo-catalyst for the removal of MV dye. The UV-Vis DRS and optical band gaps were determined to gain an insight into the semiconductor behaviors of 1–5. As shown in Supplementary Figures S19–S23, strong and broad-range ultraviolet light absorption in the 220–340 nm range was observed for 1–5, attributed to π→π* transitions in the ligands or ligand-to-metal charge transfer (LMCT) (Othong et al., 2017). In addition, a relatively weak absorption band was observed at 440–620 nm for 1–5; the d→d spin-allowed transition of Co^2+^ (d^7^) ions (Liu et al., 2014). Using the Kubelka-Munk function, the band gap energies (*E*g) were estimated to be 3.30 eV, 3.10 eV, 3.60 eV, 3.20 eV, and 3.45 eV for 1–5, respectively, falling in the UV region (<3.1 eV for the visible region) (Sarkar et al., 2020). Based on the above structural analysis, the higher-activity sites of the coordinated unit in 2 may contribute to its distinguished photocatalytic properties. To quantify the stability of 2 after reaction with the dye, PXRD was performed (Supplementary Figure S24). The results indicate that the structure of two remained unchanged after dye degradation, thus proving it to be a stable photocatalyst.

## Conclusion

In summary, we have successfully synthesized five Co-MOFs exhibiting sheet-like frameworks using a solvothermal method at mild conditions (130 C). The structures of the Co-MOFs are discussed and compared, and the results indicate that three types of networks are formed. Among the five MOFs, four exhibits superior electrocatalytic activity performance for the OER, with a low overpotential and a small Tafel slope. The ESCA, the electrolyte resistance (R_s_), and the charge transfer resistance (R_ct_) for four are 180 cm^−2^, 0.22 Ω, and 1.21Ω, respectively. Moreover, four exhibits long-term durability for at least 54,000 s at a current density of 10 mA cm^−2^, highlighting its robust stability in alkaline conditions. The strategy of constructing 2D layered MOFs with empty active sites is a promising method for the rational design and synthesis of high-performance OER electrocatalysts. The photocatalytic performance of two suggests that it may be a promising photocatalyst.

## Data Availability

The original contributions presented in the study are included in the article/Supplementary Material, further inquiries can be directed to the corresponding authors. CCDC 2195002, 2195003, 2194999, 2195001, 2195000, for MOFs 1–5 include the crystal data for this paper. These data can be freely accessed *via* www.ccdc.cam.ac.uk, or by emailing data_request@ccdc.cam.ac.uk, or by contacting The Cambridge Crystallographic Data Centre, 12 Union Road, Cambridge CB2 1EZ, United Kingdom; fax: + 44 1223336033.
