# Supplementary material for: Design of five two-dimensional Co-metal-organic frameworks for oxygen evolution reaction and dye degradation properties
Source: Front Chem. 2022 Nov 10;10:1044313. doi: 10.3389/fchem.2022.1044313 (PMC9685625; doi:10.3389/fchem.2022.1044313)

checkCIF/PLATON report

You have not supplied any structure factors. As a result the full set of tests cannot be run.

THIS REPORT IS FOR GUIDANCE ONLY. IF USED AS PART OF A REVIEW PROCEDURE FOR PUBLICATION, IT SHOULD NOT REPLACE THE EXPERTISE OF AN EXPERIENCED CRYSTALLOGRAPHIC REFEREE.

No syntax errors found. CIF dictionary Interpreting this report

Datablock: 6_0m

Bond precision:	C-C = 0.0064 A		Wavelength=0.71073
Cell:	a=15.4621(12)	b=11.9109(9)	c=17.5891(16)
	alpha=90	beta=102.271(3)	gamma=90
Temperature:	150 K		

	Calculated	Reported
Volume	3165.3(4)	3165.3(4)
Space group	P 21/n	P 1 21/n 1
Hall group	-P 2yn	-P 2yn
Moiety formula	C38 H28 Co N4 O8	C38 H28 Co N4 O8
Sum formula	C38 H28 Co N4 O8	C38 H28 Co N4 O8
Mr	727.57	727.57
Dx,g cm-3	1.527	1.527
Z	4	4
Mu (mm-1)	0.607	0.607
F000	1500.0	1500.0
F000'	1502.07	
h,k,lmax	19,14,22	19,14,22
Nref	6569	6451
Tmin,Tmax	0.916,0.953	0.590,0.745
Tmin'	0.891	

Correction method= # Reported T Limits: Tmin=0.590 Tmax=0.745
AbsCorr = MULTI-SCAN

Data completeness= 0.982 Theta(max)= 26.522

R(reflections)= 0.0614(4174) wR2(reflections)= 0.1864(6451)

S = 1.058 Npar= 461

The following ALERTS were generated. Each ALERT has the format
test-name_ALERT_alert-type_alert-level.
Click on the hyperlinks for more details of the test.

Alert level B

```
PLAT232_ALERT_2_B Hirshfeld Test Diff (M-X)  Co1      --O7       .       14.0 s.u.
PLAT420_ALERT_2_B D-H Bond Without Acceptor  O7       --H7A      .     Please Check
```

Alert level C

```
PLAT242_ALERT_2_C Low    'MainMol' Ueq as Compared to Neighbors of        Co1 Check
PLAT341_ALERT_3_C Low Bond Precision on  C-C Bonds ...............    0.00638 Ang.
PLAT415_ALERT_2_C Short Inter D-H..H-X       H7A      ..H21      .       2.14 Ang.
                                     3/2-x,-1/2+y,3/2-z  =      2_646 Check
PLAT415_ALERT_2_C Short Inter D-H..H-X       H7A      ..H22      .       2.01 Ang.
                                     3/2-x,-1/2+y,3/2-z  =      2_646 Check
```

Alert level G

```
PLAT004_ALERT_5_G Polymeric Structure Found with Maximum Dimension          2 Info
PLAT007_ALERT_5_G Number of Unrefined Donor-H Atoms .............          2 Report
PLAT012_ALERT_1_G No       _shelx_res_checksum Found in CIF ......     Please Check
PLAT794_ALERT_5_G Tentative Bond Valency for Co1       (II)      .       1.95 Info
PLAT941_ALERT_3_G Average HKL Measurement Multiplicity ..........        2.9 Low
```

0 **ALERT level A** = Most likely a serious problem - resolve or explain
2 **ALERT level B** = A potentially serious problem, consider carefully
4 **ALERT level C** = Check. Ensure it is not caused by an omission or oversight
5 **ALERT level G** = General information/check it is not something unexpected

1 ALERT type 1 CIF construction/syntax error, inconsistent or missing data
5 ALERT type 2 Indicator that the structure model may be wrong or deficient
2 ALERT type 3 Indicator that the structure quality may be low
0 ALERT type 4 Improvement, methodology, query or suggestion
3 ALERT type 5 Informative message, check

It is advisable to attempt to resolve as many as possible of the alerts in all categories. Often the minor alerts point to easily fixed oversights, errors and omissions in your CIF or refinement strategy, so attention to these fine details can be worthwhile. In order to resolve some of the more serious problems it may be necessary to carry out additional measurements or structure refinements. However, the purpose of your study may justify the reported deviations and the more serious of these should normally be commented upon in the discussion or experimental section of a paper or in the "special_details" fields of the CIF. checkCIF was carefully designed to identify outliers and unusual parameters, but every test has its limitations and alerts that are not important in a particular case may appear. Conversely, the absence of alerts does not guarantee there are no aspects of the results needing attention. It is up to the individual to critically assess their own results and, if necessary, seek expert advice.

Publication of your CIF in IUCr journals

A basic structural check has been run on your CIF. These basic checks will be run on all CIFs submitted for publication in IUCr journals (*Acta Crystallographica*, *Journal of Applied Crystallography*, *Journal of Synchrotron Radiation*); however, if you intend to submit to *Acta Crystallographica Section C* or *E* or *IUCrData*, you should make sure that full publication checks are run on the final version of your CIF prior to submission.

Publication of your CIF in other journals

Please refer to the *Notes for Authors* of the relevant journal for any special instructions relating to CIF submission.

PLATON version of 18/05/2022; check.def file version of 17/05/2022

Datablock 6_0m - ellipsoid plot

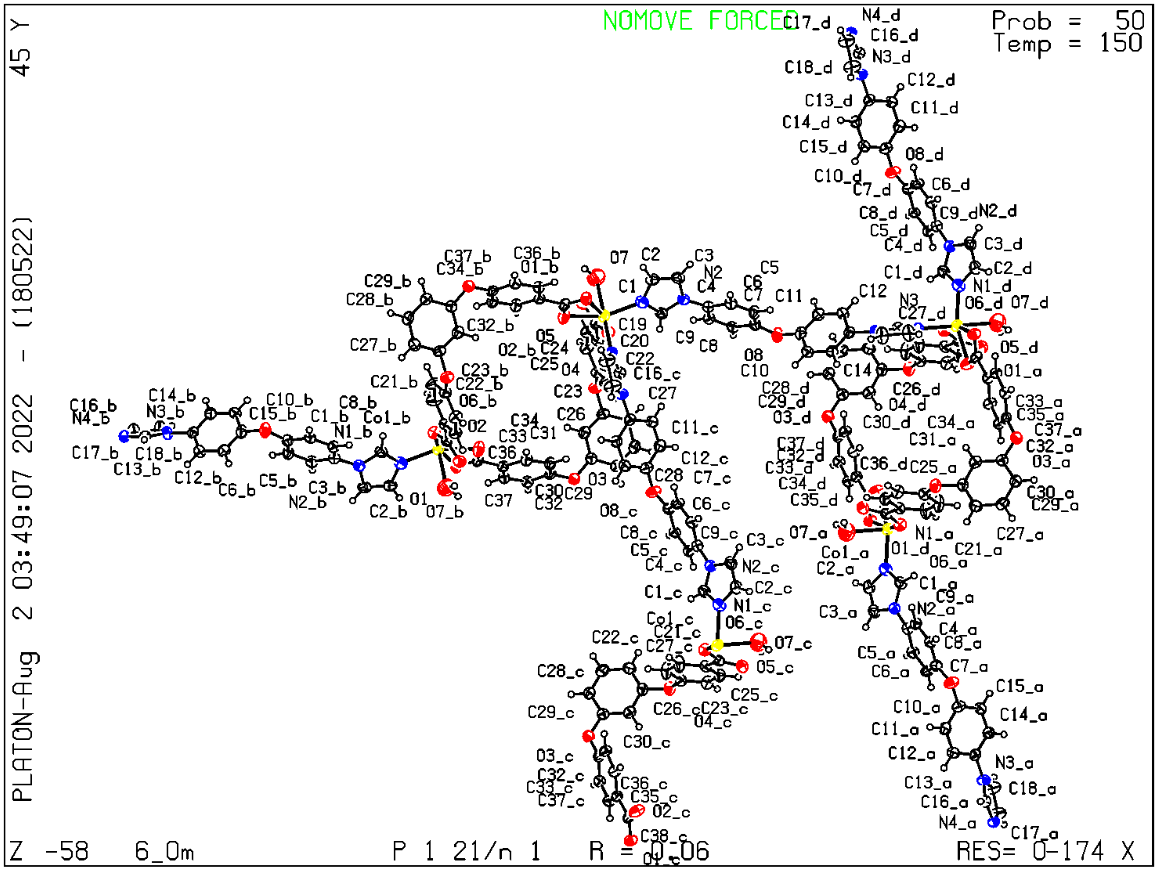

Supplement: Supplementary file 3 [file DataSheet6.PDF]
