# Supplementary material for: Design of five two-dimensional Co-metal-organic frameworks for oxygen evolution reaction and dye degradation properties
Source: Front Chem. 2022 Nov 10;10:1044313. doi: 10.3389/fchem.2022.1044313 (PMC9685625; doi:10.3389/fchem.2022.1044313)

checkCIF/PLATON report

You have not supplied any structure factors. As a result the full set of tests cannot be run.

THIS REPORT IS FOR GUIDANCE ONLY. IF USED AS PART OF A REVIEW PROCEDURE FOR PUBLICATION, IT SHOULD NOT REPLACE THE EXPERTISE OF AN EXPERIENCED CRYSTALLOGRAPHIC REFEREE.

No syntax errors found. CIF dictionary Interpreting this report

Datablock: 12_0m_sq

```
Bond precision:    C-C = 0.0052 A            Wavelength=0.71073

Cell:              a=15.8024(3)     b=42.0723(11)       c=19.6622(4)
                   alpha=90         beta=107.605(1)     gamma=90
Temperature:       170 K
```

	Calculated	Reported
Volume	12460.0(5)	12460.0(5)
Space group	P 21/n	P 1 21/n 1
Hall group	-P 2yn	-P 2yn
Moiety formula	C92 H60 Co2 N8 O12, C46 H30 Co N4 O6, C4 H9 N O, H2 O [+ solven	1.5(C92 H60 Co2 N8 O12), H2 O, C4 H9 N O
Sum formula	C142 H101 Co3 N13 O20 [+ solvent]	C142 H101 Co3 N13 O20
Mr	2486.15	2486.14
Dx,g cm-3	1.325	1.325
Z	4	4
Mu (mm-1)	0.470	0.470
F000	5140.0	5140.0
F000'	5146.31	
h,k,lmax	19,52,24	19,52,24
Nref	25598	25445
Tmin,Tmax	0.956,0.977	0.681,0.745
Tmin'	0.932	

```
Correction method= # Reported T Limits: Tmin=0.681 Tmax=0.745
AbsCorr = MULTI-SCAN

Data completeness= 0.994            Theta(max)= 26.414
```

R(reflections)= 0.0560(14952)　　wR2(reflections)= 0.1409(25445)

S = 1.011　　Npar= 1609

The following ALERTS were generated. Each ALERT has the format
test-name_ALERT_alert-type_alert-level.
Click on the hyperlinks for more details of the test.

Alert level C

PLAT241_ALERT_2_C High 'MainMol' Ueq as Compared to Neighbors of O00S Check
PLAT241_ALERT_2_C High 'MainMol' Ueq as Compared to Neighbors of O106 Check

---

Alert level G

PLAT004_ALERT_5_G Polymeric Structure Found with Maximum Dimension 2 Info
PLAT007_ALERT_5_G Number of Unrefined Donor-H Atoms .... 2 Report
PLAT012_ALERT_1_G No _shelx_res_checksum Found in CIF ...... Please Check
PLAT014_ALERT_1_G No _shelx_fab_checksum Found in CIF ...... Please Check
PLAT042_ALERT_1_G Calc. and Reported MoietyFormula Strings Differ Please Check
PLAT083_ALERT_2_G SHELXL Second Parameter in WGHT Unusually Large 6.12 Why ?
PLAT606_ALERT_4_G Solvent Accessible VOID(S) in Structure .... ! Info
PLAT720_ALERT_4_G Number of Unusual/Non-Standard Labels .... 2 Note
PLAT794_ALERT_5_G Tentative Bond Valency for Co1 (II) . 1.86 Info
PLAT794_ALERT_5_G Tentative Bond Valency for Co2 (II) . 1.92 Info
PLAT794_ALERT_5_G Tentative Bond Valency for Co3 (II) . 1.82 Info
PLAT869_ALERT_4_G ALERTS Related to the Use of SQUEEZE Suppressed ! Info
PLAT941_ALERT_3_G Average HKL Measurement Multiplicity .... 3.2 Low

---

0 **ALERT level A** = Most likely a serious problem - resolve or explain
0 **ALERT level B** = A potentially serious problem, consider carefully
2 **ALERT level C** = Check. Ensure it is not caused by an omission or oversight
13 **ALERT level G** = General information/check it is not something unexpected

3 ALERT type 1 CIF construction/syntax error, inconsistent or missing data
3 ALERT type 2 Indicator that the structure model may be wrong or deficient
1 ALERT type 3 Indicator that the structure quality may be low
3 ALERT type 4 Improvement, methodology, query or suggestion
5 ALERT type 5 Informative message, check

It is advisable to attempt to resolve as many as possible of the alerts in all categories. Often the minor alerts point to easily fixed oversights, errors and omissions in your CIF or refinement strategy, so attention to these fine details can be worthwhile. In order to resolve some of the more serious problems it may be necessary to carry out additional measurements or structure refinements. However, the purpose of your study may justify the reported deviations and the more serious of these should normally be commented upon in the discussion or experimental section of a paper or in the "special_details" fields of the CIF. checkCIF was carefully designed to identify outliers and unusual parameters, but every test has its limitations and alerts that are not important in a particular case may appear. Conversely, the absence of alerts does not guarantee there are no aspects of the results needing attention. It is up to the individual to critically assess their own results and, if necessary, seek expert advice.

Publication of your CIF in IUCr journals

A basic structural check has been run on your CIF. These basic checks will be run on all CIFs submitted for publication in IUCr journals (*Acta Crystallographica*, *Journal of Applied Crystallography*, *Journal of Synchrotron Radiation*); however, if you intend to submit to *Acta Crystallographica Section C* or *E* or *IUCrData*, you should make sure that full publication checks are run on the final version of your CIF prior to submission.

Publication of your CIF in other journals

Please refer to the *Notes for Authors* of the relevant journal for any special instructions relating to CIF submission.

PLATON version of 18/05/2022; check.def file version of 17/05/2022

Datablock 12_0m_sq - ellipsoid plot

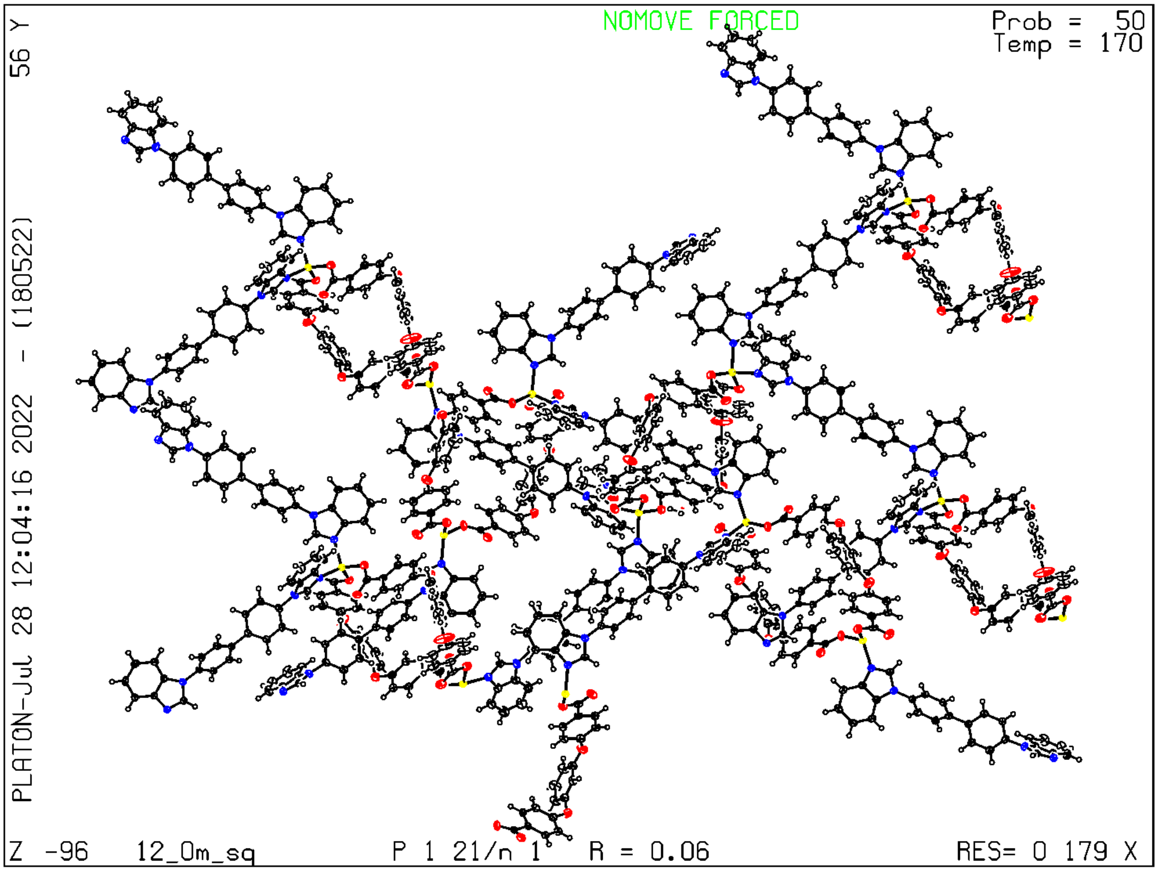

Supplement: Supplementary file 4 [file DataSheet3.PDF]
