# Supplementary material for: Design of five two-dimensional Co-metal-organic frameworks for oxygen evolution reaction and dye degradation properties
Source: Front Chem. 2022 Nov 10;10:1044313. doi: 10.3389/fchem.2022.1044313 (PMC9685625; doi:10.3389/fchem.2022.1044313)
Supplement: Supplementary file 5 [file DataSheet1.PDF]

Design of Five 2D Co-MOFs for OER and dye degradation properties

Chuanbin Fan,[a,d] Xiaoyin Zhang,[b,e] Feng Guo,[a,d] Zhiyong Xing,[a,d] Junli Wang,[a,d] Wanying Lin,[a] Jie Tan,[a] Guimei Huang,[a,c,**] Ziao Zong. [a,d,*]

a. School of Laboratory Medicine, Youjiang Medical University for Nationalities, Baise, Guangxi, 533000, P.R. China
b. Institute of Oceanographic Instrumentation, Qilu University of Technology (Shandong Academy of Sciences) Qingdao 266061, P. R. China
c. School of Chemistry and Pharmaceutical Sciences, Guangxi Normal University, Guilin 541004, People's Republic of China
d. Industrial College of biomedicine and health industry, Youjiang Medical University for Nationalities, Baise, Guangxi, 533000, P.R. China
e. Key Laboratory of Advanced Energy Materials Chemistry (Ministry of Education), College of Chemistry, Nankai University, Tianjin 300071, China

*Corresponding author: zongziao@126.com.
**Corresponding author: guimei1128@163.com.

Materials and analysis.

The N/O-donor ligands — H_2L = 1,3-bis(4′-carboxylphenoxy)benzene acid, 4,4′-bbidpe = 4,4′-bis(benzimidazol-1-yl)diphenyl ether, 4,4′-bbibp = 4,4′-bis(benzimidazol-1-yl)biphenyl, 3,5-bip = 3,5-bis(1-imidazoly)pyridine, 1,4-bimb = 1,4-bis(imidazol-1ylmethyl)benzene, and 4,4′-bidpe = 4,4′-bis(imidazolyl)diphenyl ether — were received from Jinan Henghua Sci. & Tec. Co. Ltd. The reagents and solvents were purchased from commercial suppliers. All chemicals were used without further purification. The Fourier-transform infrared (FT-IR) spectra of YMUN **1**–**5** was recorded with the samples prepared as KBr discs through the Nicolet 170SX spectrometer in the range of 4000–400 cm^{-1}. The collection of single crystal X-ray diffractometer data is in application of a Bruker APEX-II CCD device with Mo-Kα radiation (λ = 0.71073 Å). Elemental analyses (C, H, and N) were performed using a Perkin-Elmer instrument, series II Model 2400 elemental analyzer. Thermogravimetric (TG) analyses data

were recorded on a Perkin-Elmer TG-7 thermogravimetric analyzer under nitrogen conditions in the range of 30 to 1000 °C with a ramp rate of 10 °C min^{-1}. The powder X-ray diffraction (PXRD) patterns were done with an Enraf-Nonius CAD-4. The UV–*vis* absorption spectra for the dye degradation performance were recorded in application of a Shimadzu UV-2550 UV-*vis* spectrophotometer. The field-emission scanning electron microscopy (SEM, KYKU-EM8100) was used to characterize the morphologies of YMUN **2** and **4**. The BET surface areas of **2** and **4** were tested by N_2 adsorption/desorption isotherms (Micromeritics ASAP 2460 surface area).

Analysis of single crystal structure.

Single crystal of **1**–**5** was chosen under an optical microscope and quickly coated with high vacuum grease (Dow Corning Corporation), and then mounted on glass fiber for data collection. The X-ray crystallography data were collected on a Bruker APEX-II CCD diffractometer with Mo-Kα radiation (λ = 0.71073 Å) at 150/170 K. The adsorption correction of single data used multi-scan method by the SADABS-2016/2 program. [1] Their structures could be successfully saved through direct method, which were refined by the full matrix least-squares method (on F^2) to convergence through the *ShelXL* (Sheldrick, 2015) program. [2] The non-hydrogen atoms were ensured and defined by the Fourier maps.

Electrochemical measurements

The electrochemical research was used three-electrode system of CHI660e electrochemical workstation of Shanghai Chenhua Company in 1.0 M KOH (pH=13.8) electrolyte. The electrode (1 cm ×1 cm), graphite plate, and saturated calomel electrode (SCE) were employed as the working, counter, and reference electrodes. CV tests were performed at a scan rate of 10 mV s^{-1} to evaluate the catalyst performance. C_{dl} of the electrocatalysts were

measured by CV at different scan rates, and calculated according to the Equation: $C_{dl} = (j_a - j_c)/(2v)$, in which j_a and j_c correspond to the current density of anode and cathode, respectively, and v is the scan rate. The C_{dl} was used to measure the ECSA. LSV was conducted for OER polarization curves with a sweeping rate of 5 mV s^{-1}. Electrochemical impedance spectroscopy (EIS) measurements were collected with a frequency range from 0.01 Hz to 200 kHz.

Dye degradation performance. The potential of **1–5** as photo-catalysts was evaluated for organic dyes (MB/MV) at the room temperature and under 125–W Hg lamp irradiation in a photo-catalytic assessment system through a series of tests. The sample of **1–5** was washed with the related reaction solution and dried for 24 h under 60 °C, and then being ground before the dye adsorption tests. The dried crystal sample (0.020 g) was added to aqueous solution of MB (100 mL, 6 mg/L), and MV (100 mL, 6 mg/L). After stirring in the dark for 30 min to ensure the establishment of an adsorption/desorption equilibrium, the aqueous solution was stirred continuously under UV irradiation. The absorbance of the solution was measured by using a UV–vis spectrophotometer every 30 min at the maximum absorption wavelength of 664 nm for MB, and 583 nm for MV.

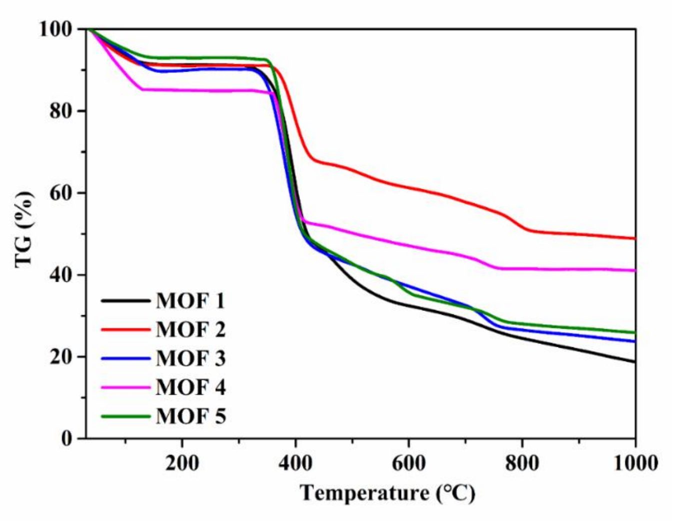

Figure S1. TG curves of **YMUN 1–5**.

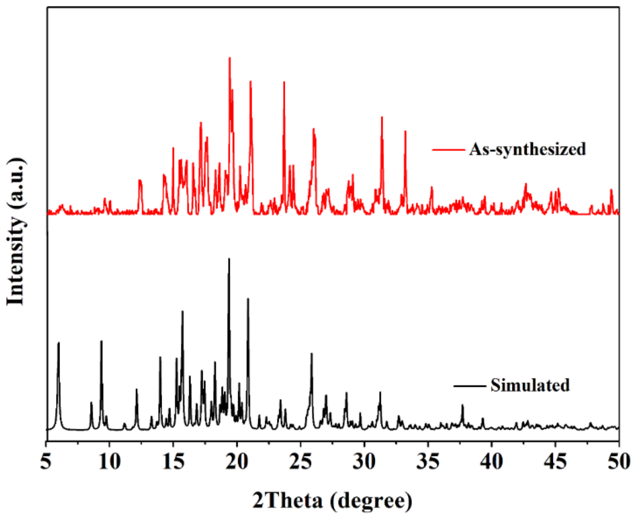

Figure S2. PXRD patterns of **1**, simulated and as-synthesized.

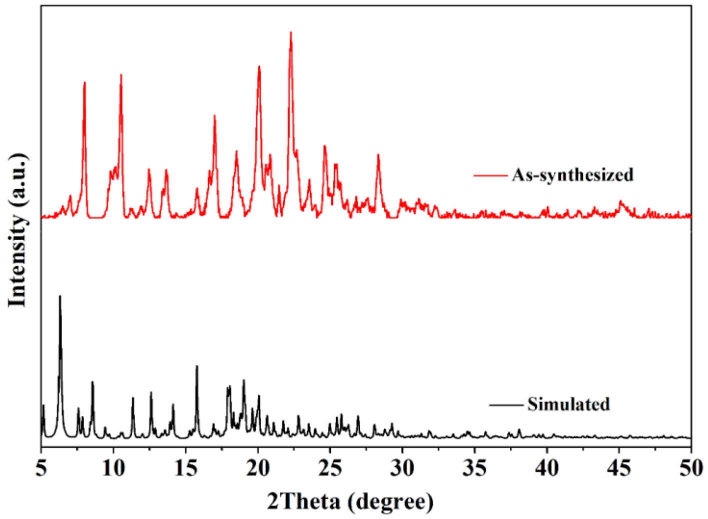

Figure S3. PXRD patterns of **2**, simulated and as-synthesized.

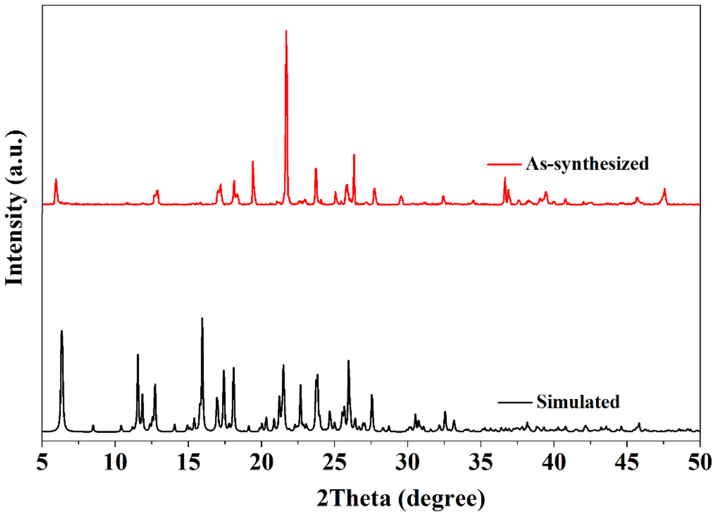

Figure S4. PXRD patterns of **3**, simulated and as-synthesized.

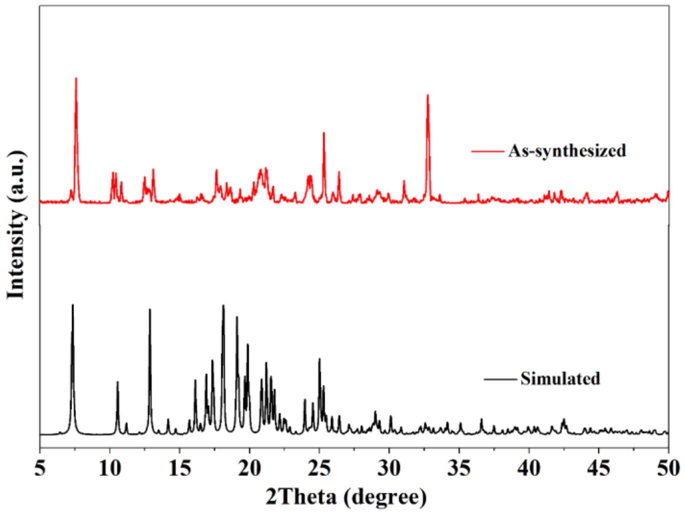

Figure S5. PXRD patterns of **4**, simulated and as-synthesized.

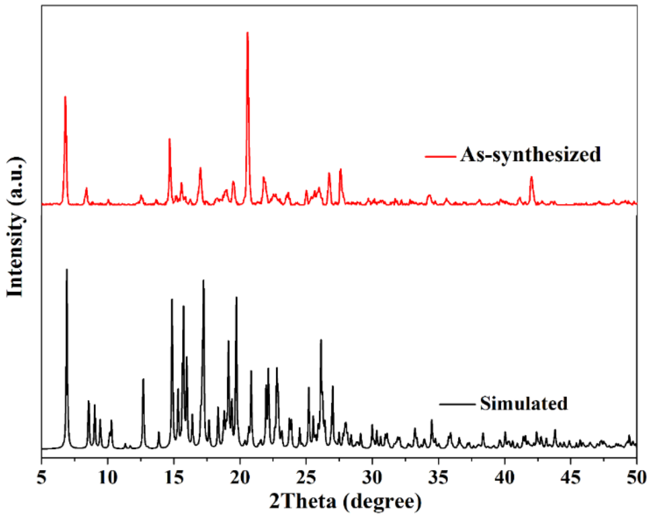

Figure S6. PXRD patterns of **5**, simulated and as-synthesized.

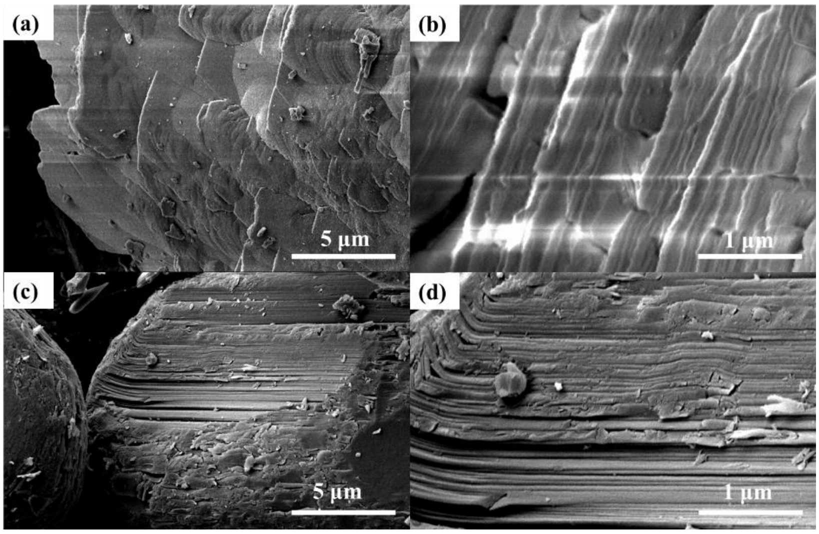

Figure S7. SEM images of (a)-(b) YMUN **2** and (c)-(d) YMUN **4**.

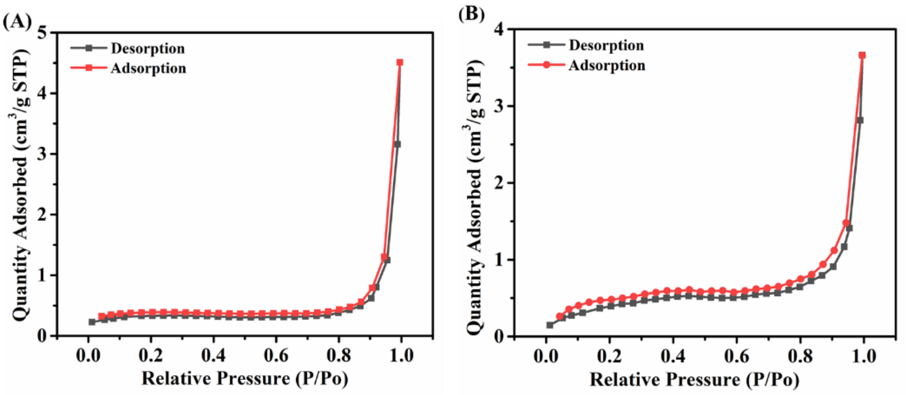

Figure S8. N_2 sorption isotherm collected at 77K for YMUN **2** and **4** under $P/P_0 = 1$.

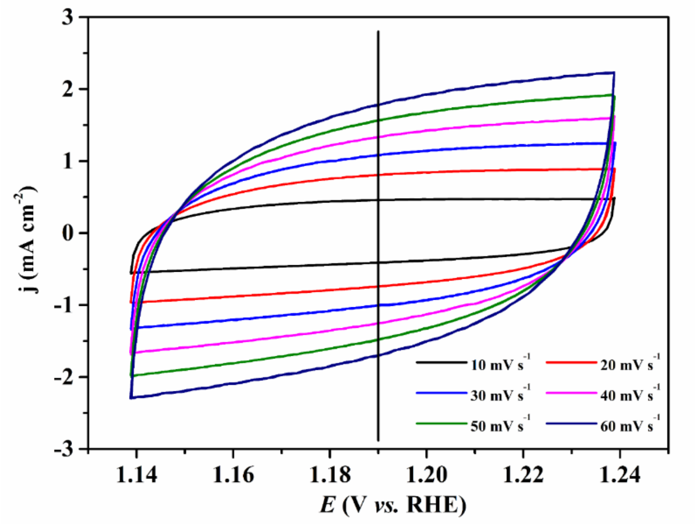

Figure S9. CV curves of **1** that operated in a non-Faradaic potential window with different scan rates

(10−60 mV s^{-1}).

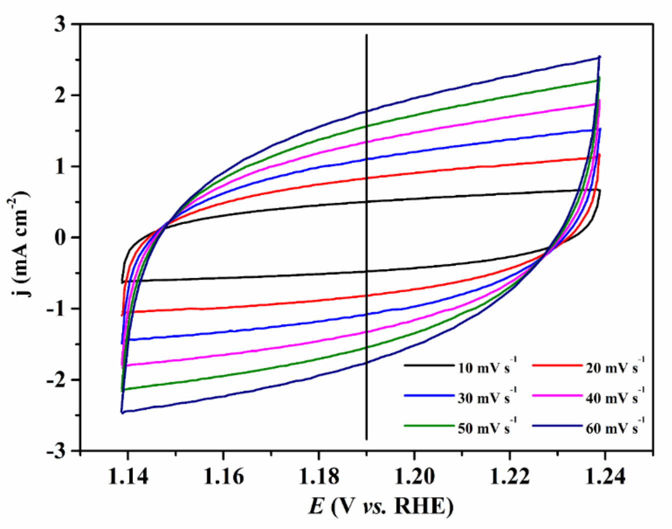

Figure S10. CV curves of **2** that operated in a non-Faradaic potential window with different scan rates (10−60 mV s^{-1}).

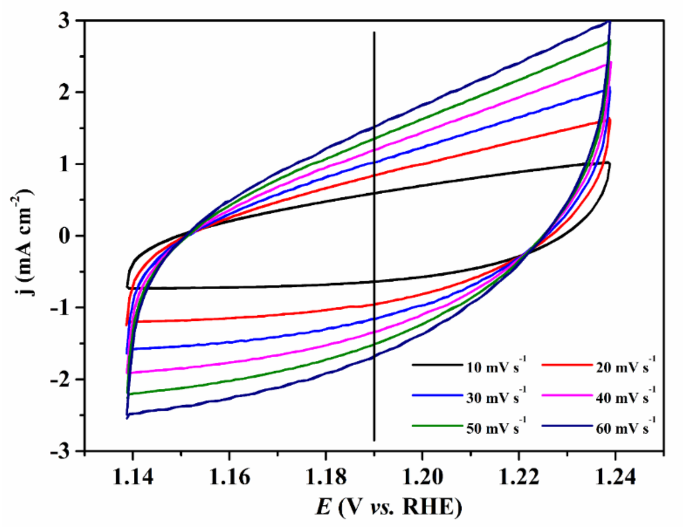

Figure S11. CV curves of **3** that operated in a non-Faradaic potential window with different scan rates (10−60 mV s^{-1}).

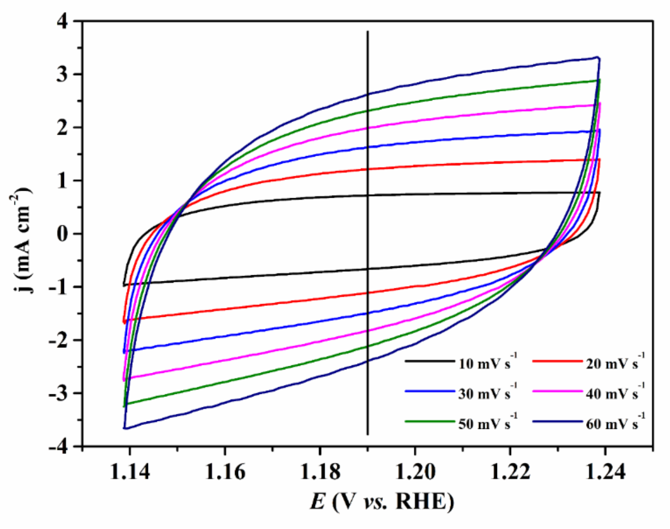

Figure S12. CV curves of **4** that operated in a non-Faradaic potential window with different scan rates (10−60 mV s^{-1}).

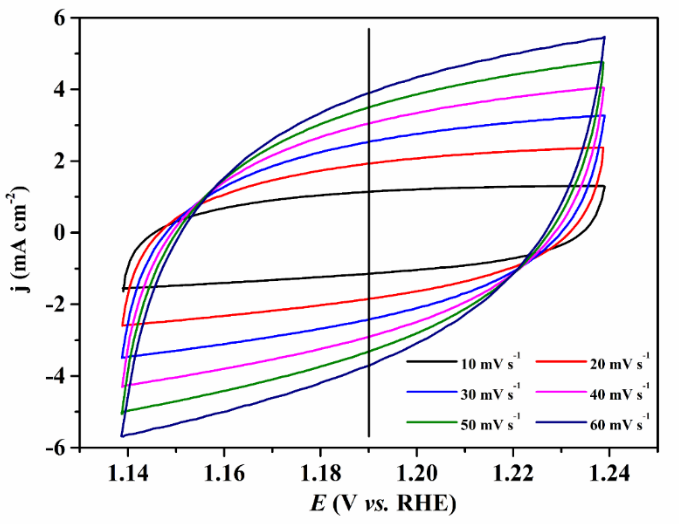

Figure S13. CV curves of **5** that operated in a non-Faradaic potential window with different scan rates (10−60 mV s^{-1}).

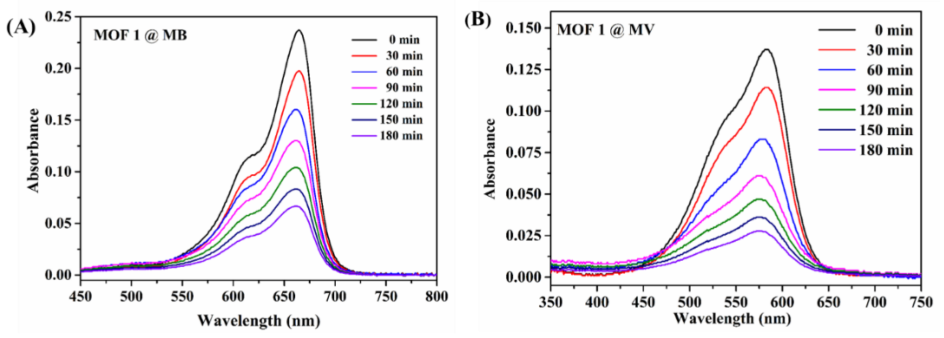

Figure S14. (A)-(B) UV-vis spectra of MB and MV dye from aqueous solutions at various time

intervals for **1**.

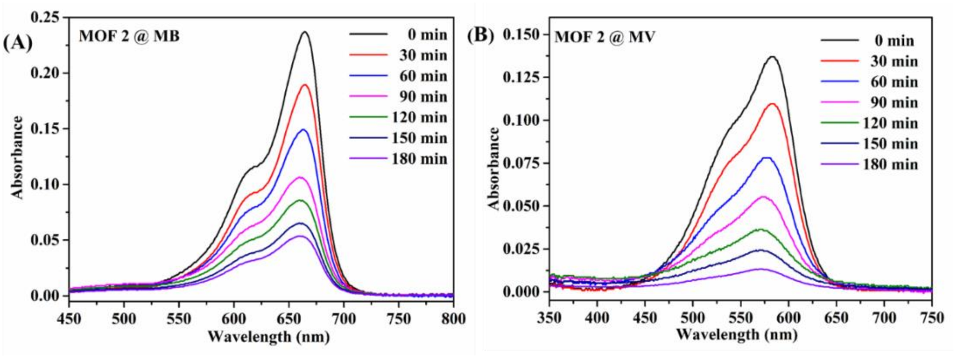

Figure S15. (A)-(B) UV-vis spectra of MB and MV dye from aqueous solutions at various time intervals for **2**.

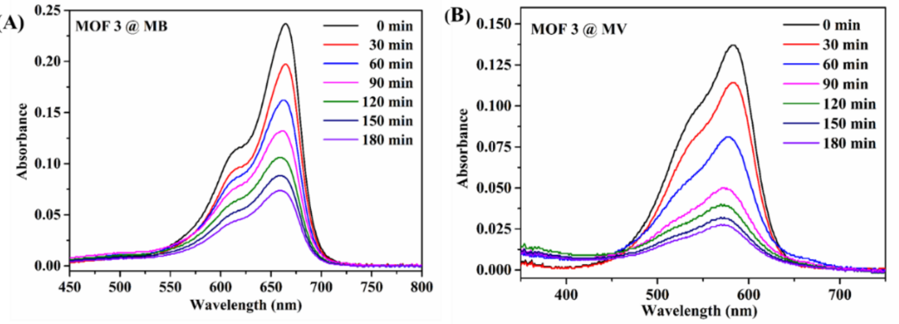

Figure S16. (A)-(B) UV-vis spectra of MB and MV dye from aqueous solutions at various time intervals for **3**.

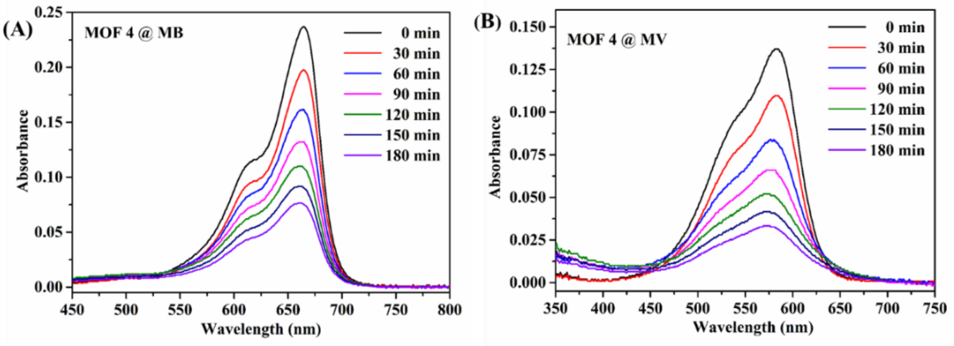

Figure S17. (A)-(B) UV-vis spectra of MB and MV dye from aqueous solutions at various time intervals for **4**.

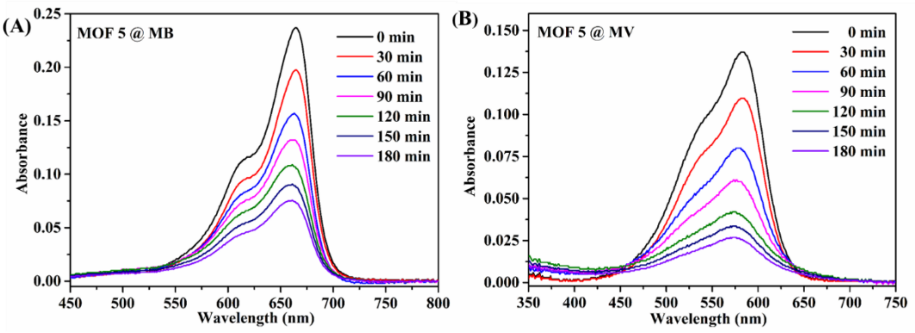

Figure S18. (A)-(B) UV-vis spectra of MB and MV dye from aqueous solutions at various time intervals for **5**.

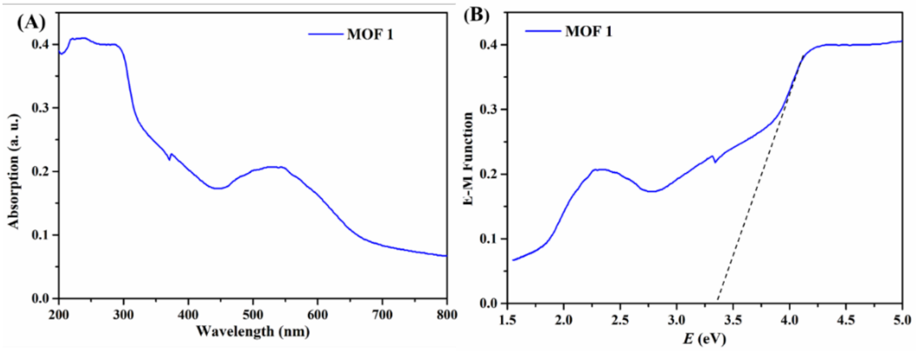

Figure S19. UV-visible diffuse reflectance spectra and Kubelka-Munk-transformed diffuse reflectance spectra of **1**.

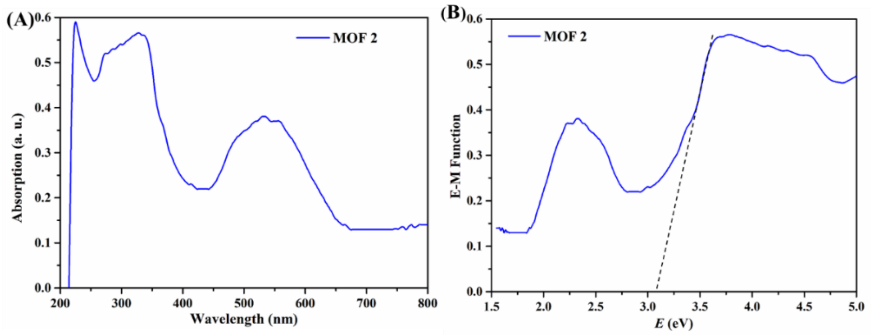

Figure S20. UV-visible diffuse reflectance spectra and Kubelka-Munk-transformed diffuse reflectance spectra of **2**.

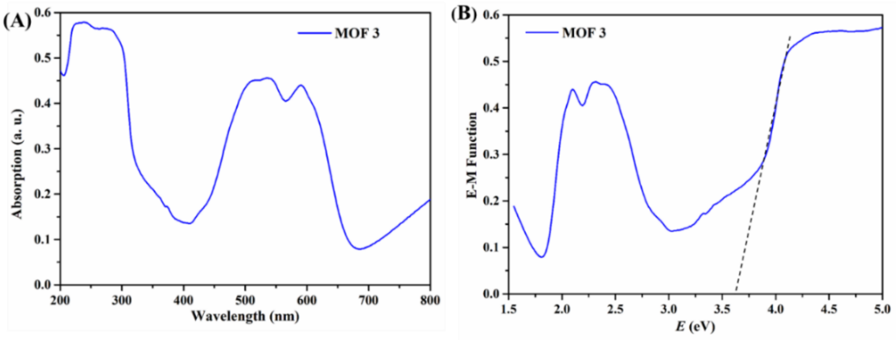

Figure S21. UV-visible diffuse reflectance spectra and Kubelka-Munk-transformed diffuse reflectance spectra of **3**.

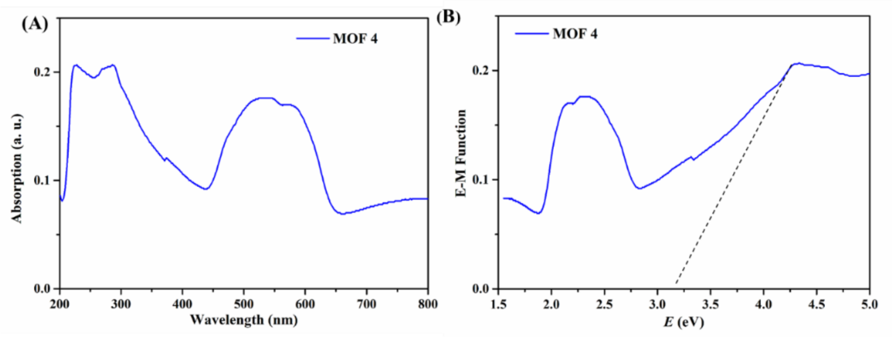

Figure S22. UV-visible diffuse reflectance spectra and Kubelka-Munk-transformed diffuse reflectance spectra of **4**.

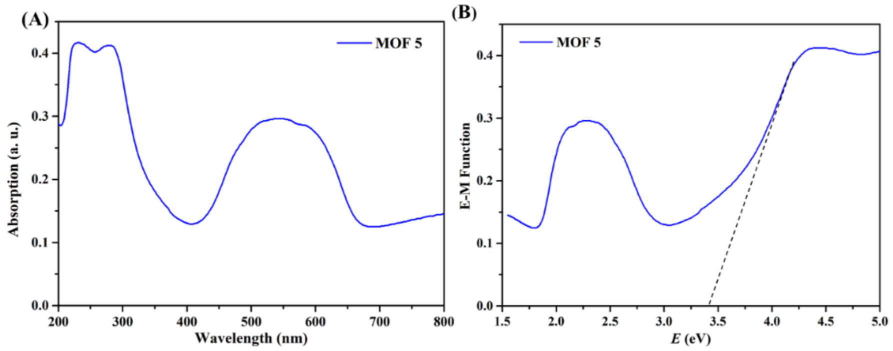

Figure S23. UV-visible diffuse reflectance spectra and Kubelka-Munk-transformed diffuse reflectance spectra of **5**.

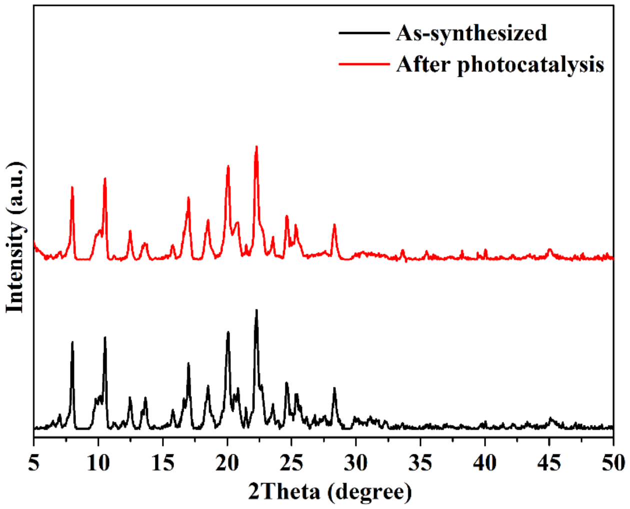

Figure S24. PXRD patterns of **2** before and after photocatalysis.

Table S1 Selected bond lengths (Å) and angles (°) for **YMUN 1–5**.

**YMUN 1**			
Co1—O2	Co1—O2	Co1—O2	Co1—O2
Co1—O7^{i}	Co1—O7^{i}	Co1—O7^{i}	Co1—O7^{i}
Co1—O6^{i}	Co1—O6^{i}	Co1—O6^{i}	Co1—O6^{i}
O2—Co1—O7^{i}	O2—Co1—O7^{i}	O2—Co1—O7^{i}	O2—Co1—O7^{i}
O2—Co1—O6^{i}	O2—Co1—O6^{i}	O2—Co1—O6^{i}	O2—Co1—O6^{i}
O2—Co1—N4^{ii}	O2—Co1—N4^{ii}	O2—Co1—N4^{ii}	O2—Co1—N4^{ii}
O2—Co1—O1	O2—Co1—O1	O2—Co1—O1	O2—Co1—O1
O2—Co1—N1	O2—Co1—N1	O2—Co1—N1	O2—Co1—N1
O2—Co1—C1^{i}	O2—Co1—C1^{i}	O2—Co1—C1^{i}	O2—Co1—C1^{i}
N4^{ii}—Co1—O1	N4^{ii}—Co1—O1	N4^{ii}—Co1—O1	N4^{ii}—Co1—O1
N4^{ii}—Co1—C1^{i}	N4^{ii}—Co1—C1^{i}	N4^{ii}—Co1—C1^{i}	N4^{ii}—Co1—C1^{i}
O1—Co1—O7^{i}	O1—Co1—O7^{i}	O1—Co1—O7^{i}	O1—Co1—O7^{i}
O1—Co1—C1^{i}	O1—Co1—C1^{i}	O1—Co1—C1^{i}	O1—Co1—C1^{i}
Co1—O1—H1A	Co1—O1—H1A	Co1—O1—H1A	Co1—O1—H1A
Symmetry codes: (i) -x+2, -y, -z+1; (ii) x+1/2, -y+3/2, z+1/2; (iii) x-1/2, -y+3/2, z-1/2.			
**YMUN 2**			
Co1—O1	1.958 (2)	Co2—N8^{i}	2.051 (3)
Co1—O16	1.952 (2)	Co2—N4^{ii}	2.048 (3)
Co1—N1	2.027 (3)	Co3—O14^{iii}	1.956 (2)

Co1—N5	2.021 (3)	Co3—O9	1.946 (2)
Co2—O7	1.985 (2)	Co3—N12	2.037 (3)
Co2—O6	1.942 (2)	Co3—N10^{iv}	2.060 (3)
O1—Co1—N1	117.96 (10)	O7—Co2—N8^{i}	117.33 (10)
O1—Co1—N5	120.64 (10)	O7—Co2—N4^{ii}	96.83 (11)
O16—Co1—O1	107.84 (10)	O6—Co2—O7	128.85 (10)
O16—Co1—N1	99.56 (10)	O6—Co2—N8^{i}	102.72 (10)
O16—Co1—N5	109.74 (11)	O6—Co2—N4^{ii}	111.96 (11)
N5—Co1—N1	98.97 (11)	N4^{ii}—Co2—N8^{i}	93.28 (11)
O14^{iii}—Co3—N12	109.12 (10)	O9—Co3—N12	120.74 (11)
O14^{iii}—Co3—N10^{iv}	96.88 (10)	O9—Co3—N10^{iv}	112.10 (10)
O9—Co3—O14^{iii}	111.27 (10)	N12—Co3—N10^{iv}	103.90 (10)

Symmetry codes: (i) -x+5/2, y+1/2, -z+5/2; (ii) -x+3/2, y+1/2, -z+3/2; (iii) -x+1, -y+2, -z+1; (iv) x-1/2, -y+3/2, z-1/2; (v) -x+5/2, y-1/2, -z+5/2; (vi) -x+3/2, y-1/2, -z+3/2; (vii) x+1/2, -y+3/2, z+1/2.

YMUN 3

Co1—O5^{i}	1.9821 (15)	Co1—N1	2.0100 (18)
Co1—O2	1.9520 (15)	Co1—N4^{ii}	2.0300 (17)
O5^{i}—Co1—N1	107.10 (7)	O2—Co1—N4^{ii}	94.06 (7)
O5^{i}—Co1—N4^{ii}	116.81 (7)	N1—Co1—N4^{ii}	113.36 (7)
O2—Co1—O5^{i}	113.99 (7)	O2—Co1—N1	111.24 (7)

Symmetry codes: (i) -x+1, -y, -z+1; (ii) x, y-1, z; (iii) x, y+1, z.

YMUN 4

Co1—O1	2.020 (4)	Co1—N4^{ii}	2.041 (4)
Co1—O5^{i}	2.020 (4)	Co1—N1	2.031 (4)
O1—Co1—N4^{ii}	113.18 (17)	O5^{i}—Co1—N4^{ii}	95.95 (16)
O1—Co1—N1	97.43 (16)	O5^{i}—Co1—N1	111.59 (17)
O5^{i}—Co1—O1	133.29 (16)	N1—Co1—N4^{ii}	102.29 (17)

Symmetry codes: (i) -x, y+1/2, -z+3/2; (ii) -x+3, y+1/2, -z+3/2; (iii) -x, y-1/2, -z+3/2; (iv) -x+3, y-1/2, -z+3/2.

YMUN 5

Co1—O1^{i}	2.279 (3)	Co1—N4^{ii}	2.086 (3)
Co1—O6	2.053 (3)	Co1—N1	2.070 (4)
Co1—O2^{i}	2.078 (3)	Co1—O7	2.193 (4)
O6—Co1—O1^{i}	162.05 (11)	O6—Co1—O7	92.18 (14)
O6—Co1—O2^{i}	101.92 (11)	O2^{i}—Co1—O1^{i}	60.25 (10)
O6—Co1—N4^{ii}	90.21 (12)	O2^{i}—Co1—N4^{ii}	97.41 (13)
O6—Co1—N1	93.61 (12)	O2^{i}—Co1—O7	81.77 (15)
N4^{ii}—Co1—O1^{i}	94.12 (11)	N1—Co1—O1^{i}	102.99 (12)

N4^{ii}—Co1—O7	177.58 (14)	N1—Co1—O2^{i}	157.97 (13)
N1—Co1—N4^{ii}	98.06 (14)	O7—Co1—O1^{i}	83.50 (13)
N1—Co1—O7	82.13 (15)		

Symmetry codes: (i) -x+1, -y, -z+1; (ii) x-1/2, -y+3/2, z-1/2; (iii) x+1/2, -y+3/2, z+1/2.

Table S2 The BET surface area, pore volume and average pore of YMUNs **2** and **4**

Samples	BET surface area (m^2/g)	Pore volume (cm^3/g)	Average pore diameter (nm)
MOF **2**	1.50	0.007	18.7
MOF **4**	1.55	0.006	14.6

References

1. Sheldrick, G. M. SADABS, Program for Empirical Absorption Correction for Area Detector Data; University of Gottingen: Gottingen, Germany, **1996**.

2. Sheldrick, G. M. Acta Crystallogr. C Sect. Struct. Chem. **2015**, *71*, 3.
