# Supplementary material for: Design of five two-dimensional Co-metal-organic frameworks for oxygen evolution reaction and dye degradation properties
Source: Front Chem. 2022 Nov 10;10:1044313. doi: 10.3389/fchem.2022.1044313 (PMC9685625; doi:10.3389/fchem.2022.1044313)

checkCIF/PLATON report

You have not supplied any structure factors. As a result the full set of tests cannot be run.

THIS REPORT IS FOR GUIDANCE ONLY. IF USED AS PART OF A REVIEW PROCEDURE FOR PUBLICATION, IT SHOULD NOT REPLACE THE EXPERTISE OF AN EXPERIENCED CRYSTALLOGRAPHIC REFEREE.

No syntax errors found.   CIF dictionary   Interpreting this report

Datablock: 0604zghy14_0m

```
Bond precision:   C-C = 0.0074 A          Wavelength=0.71073

Cell:              a=7.3088(9)     b=24.077(4)        c=16.768(2)
                   alpha=90        beta=92.819(4)     gamma=90
Temperature:       170 K

                 Calculated                   Reported
Volume           2947.2(7)                    2947.1(7)
Space group      P 21/c                       P 1 21/c 1
Hall group       -P 2ybc                      -P 2ybc
Moiety formula   C34 H26 Co N4 O6             C34 H26 Co N4 O6
Sum formula      C34 H26 Co N4 O6             C34 H26 Co N4 O6
Mr               645.52                       645.52
Dx,g cm-3        1.455                        1.455
Z                4                            4
Mu (mm-1)        0.637                        0.637
F000             1332.0                       1332.0
F000'            1333.95
h,k,lmax         9,30,21                      9,30,21
Nref             6130                         5872
Tmin,Tmax        0.941,0.969                  0.617,0.745
Tmin'            0.909

Correction method= # Reported T Limits: Tmin=0.617 Tmax=0.745
AbsCorr = MULTI-SCAN

Data completeness= 0.958           Theta(max)= 26.519

                                                       wR2(reflections)=
R(reflections)= 0.0728( 3556)                          0.1640( 5872)

S = 1.082                    Npar= 406
```

The following ALERTS were generated. Each ALERT has the format
test-name_ALERT_alert-type_alert-level.
Click on the hyperlinks for more details of the test.

Alert level C

```
PLAT029_ALERT_3_C _diffrn_measured_fraction_theta_full value Low .      0.973 Why?
PLAT230_ALERT_2_C Hirshfeld Test Diff for    O5       --C20      .        5.5 s.u.
PLAT341_ALERT_3_C Low Bond Precision on  C-C Bonds ...............    0.00737 Ang.
```

Alert level G

```
PLAT004_ALERT_5_G Polymeric Structure Found with Maximum Dimension          2 Info
PLAT012_ALERT_1_G No       _shelx_res_checksum Found in CIF ......     Please Check
PLAT083_ALERT_2_G SHELXL Second Parameter in WGHT  Unusually Large       8.53 Why ?
PLAT232_ALERT_2_G Hirshfeld Test Diff (M-X)  Co1       --O5_b     .       6.5 s.u.
PLAT794_ALERT_5_G Tentative Bond Valency for Co1       (II)       .      1.93 Info
PLAT804_ALERT_5_G Number of ARU-Code Packing Problem(s) in PLATON          60 Info
PLAT941_ALERT_3_G Average HKL Measurement Multiplicity ...........        3.7 Low
```

0 **ALERT level A** = Most likely a serious problem - resolve or explain
0 **ALERT level B** = A potentially serious problem, consider carefully
3 **ALERT level C** = Check. Ensure it is not caused by an omission or oversight
7 **ALERT level G** = General information/check it is not something unexpected

1 ALERT type 1 CIF construction/syntax error, inconsistent or missing data
3 ALERT type 2 Indicator that the structure model may be wrong or deficient
3 ALERT type 3 Indicator that the structure quality may be low
0 ALERT type 4 Improvement, methodology, query or suggestion
3 ALERT type 5 Informative message, check

It is advisable to attempt to resolve as many as possible of the alerts in all categories. Often the minor alerts point to easily fixed oversights, errors and omissions in your CIF or refinement strategy, so attention to these fine details can be worthwhile. In order to resolve some of the more serious problems it may be necessary to carry out additional measurements or structure refinements. However, the purpose of your study may justify the reported deviations and the more serious of these should normally be commented upon in the discussion or experimental section of a paper or in the "special_details" fields of the CIF. checkCIF was carefully designed to identify outliers and unusual parameters, but every test has its limitations and alerts that are not important in a particular case may appear. Conversely, the absence of alerts does not guarantee there are no aspects of the results needing attention. It is up to the individual to critically assess their own results and, if necessary, seek expert advice.

Publication of your CIF in IUCr journals

A basic structural check has been run on your CIF. These basic checks will be run on all CIFs submitted for publication in IUCr journals (*Acta Crystallographica*, *Journal of Applied Crystallography*, *Journal of Synchrotron Radiation*); however, if you intend to submit to *Acta Crystallographica Section C* or *E* or *IUCrData*, you should make sure that full publication checks are run on the final version of your CIF prior to submission.

Publication of your CIF in other journals

Please refer to the *Notes for Authors* of the relevant journal for any special instructions relating to CIF submission.

PLATON version of 18/05/2022; check.def file version of 17/05/2022

Datablock 0604zghy14_0m - ellipsoid plot

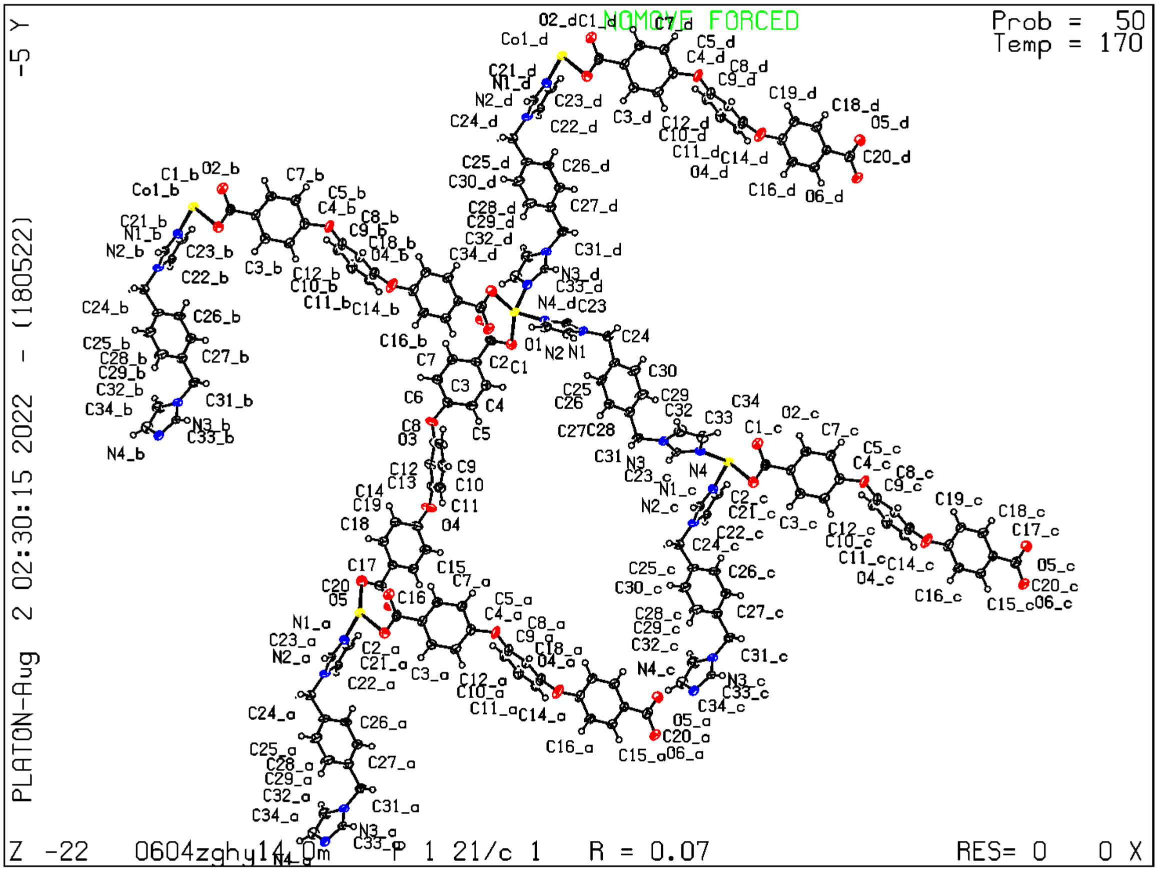

Supplement: Supplementary file 6 [file DataSheet5.PDF]
